# Metabolic profiling of prostate cancer in skeletal microenvironments identifies G6PD as a key mediator of growth and survival

**DOI:** 10.1126/sciadv.abf9096

**Published:** 2022-02-25

**Authors:** Jessica Whitburn, Srinivasa R. Rao, Emma V. Morris, Sho Tabata, Akiyoshi Hirayama, Tomoyoshi Soga, James R. Edwards, Zeynep Kaya, Charlotte Palmer, Freddie C. Hamdy, Claire M. Edwards

**Affiliations:** 1 Nuffield Department of Surgical Sciences, University of Oxford, Oxford, UK.; 2 Institute for Advanced Biosciences, Keio University, Yamagata, Japan.; 3 Nuffield Department of Orthopaedics, Rheumatology and Musculoskeletal Sciences, University of Oxford, Oxford, UK.

## Abstract

The spread of cancer to bone is invariably fatal, with complex cross-talk between tumor cells and the bone microenvironment responsible for driving disease progression. By combining in silico analysis of patient datasets with metabolomic profiling of prostate cancer cells cultured with bone cells, we demonstrate the changing energy requirements of prostate cancer cells in the bone microenvironment, identifying the pentose phosphate pathway (PPP) as elevated in prostate cancer bone metastasis, with increased expression of the PPP rate-limiting enzyme glucose-6-phosphate dehydrogenase (G6PD) associated with a reduction in progression-free survival. Genetic and pharmacologic manipulation demonstrates that G6PD inhibition reduces prostate cancer growth and migration, associated with changes in cellular redox state and increased chemosensitivity. Genetic blockade of G6PD in vivo results in reduction of tumor growth within bone. In summary, we demonstrate the metabolic plasticity of prostate cancer cells in the bone microenvironment, identifying the PPP and G6PD as metabolic targets for the treatment of prostate cancer bone metastasis.

## Teaser

Metabolic changes in the prostate cancer–bone microenvironment promote tumor growth and survival.

## INTRODUCTION

Prostate cancer is the second leading cause of cancer-related death in men. While overall 5-year survival rates for prostate cancer are 97.8%, in metastatic disease this falls to only 30% (*1*). The mainstay of treatment for metastatic prostate cancer since the 1940s has been androgen deprivation therapy (ADT), but the cancer typically becomes resistant with a median survival of approximately 3 years (*2*). In the past few years, several new treatment options (e.g., enzalutamide and abiraterone) have been approved, but these have led to only modest overall survival benefits (*3*). Docetaxel is generally considered the first-line chemotherapy for metastatic prostate cancer; however, only approximately 50% of patients respond, and most eventually develop resistance (*4*).

Prostate cancer commonly metastasizes to the bone, with skeletal involvement present in approximately 90% of patients with metastatic disease (*5*). The exact reasons for this affinity remain poorly understood; however, once prostate cancer has colonized the bone, a vicious cycle develops where cancer cells secrete factors that dysregulate bone remodeling, leading to the release of growth factors from bone that further promote prostate cancer growth and survival. The bone disease seen is typically classed as osteoblastic/osteosclerotic, reflecting the balance of bone remodeling that is tipped in favor of bone deposition. Bone remodeling is a highly energy-consuming process; however, the metabolic changes that underpin malignant bone disease have not been well characterized.

The normal human prostate has a unique metabolism compared to other tissues, with high zinc levels leading to a truncated tricarboxylic acid (TCA) cycle allowing the accumulation of extraordinarily high levels of citrate (*6*). A hallmark of malignant transformation of the prostate is the loss of ability to concentrate zinc, releasing inhibition of the TCA cycle. Most cancers become less energy efficient as they undergo malignant change (*7*, *8*); however, prostate cancer cells become more energy efficient, transforming from citrate-secreting cells to citrate-oxidizing cells, allowing increased flow through the TCA cycle and increased oxidative phosphorylation (OXPHOS). This metabolic transformation is an early event in preparation for the progression to malignancy (*9*); however, the metabolic changes induced by the bone metastatic environment remain unclear. Elucidating how metabolic imbalance drives prostate cancer bone metastasis will reveal new targets for the treatment of this fatal advanced disease stage. In this study, we investigated the metabolic changes seen in metastatic prostate cancer to bone, using a combination of in vitro, in vivo, and in silico approaches.

The pentose phosphate pathway (PPP) and in particular its rate-limiting enzyme glucose-6-phosphate dehydrogenase (G6PD) were found to be consistently up-regulated and correlated with patient outcome. Bone marrow stromal cells were found to up-regulate G6PD expression via interleukin-6 (IL-6) secretion, and targeting G6PD either pharmacologically or genetically could inhibit prostate cancer proliferation, migration, mesenchymal phenotype, and sensitivity to chemotherapy. Overall, our study suggests that inhibition of G6PD activity may be developed as a therapeutic strategy for bone metastatic prostate cancer in the future.

## RESULTS

### The bone microenvironment alters the metabolic profile of prostate cancer cells

**Fig. 1.**
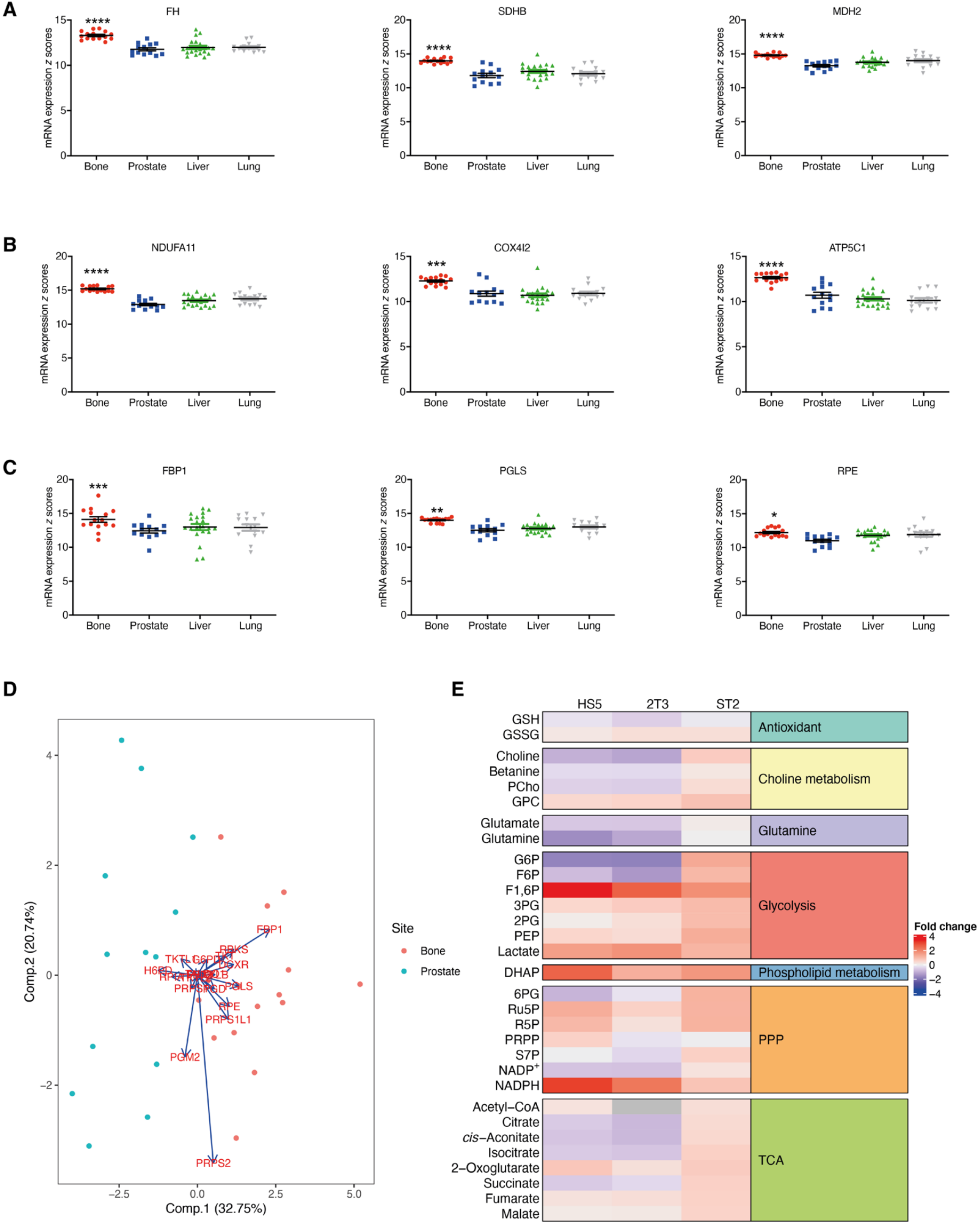
The bone microenvironment alters the metabolic profile of prostate cancer cells. In silico analysis of primary and metastatic prostate cancer, comparing three genes from each of the TCA (**A**), OXPHOS (**B**), and PPP (**C**) pathways in primary prostate cancer and bone, liver, and lung metastatic sites. *P < 0.05, **P < 0.01, ***P < 0.001, ****P < 0.0001 as compared to primary prostate cancer. Statistical test used: one-way analysis of variance (ANOVA) with post hoc Tukey’s test. Error bars = SEM. (**D**) Principal components analysis plot of PPP genes in bone metastatic and primary prostate cancer. (**E**) PC3 cells were cocultured for 24 hours with HS5, ST2, or 2T3 bone stromal cells, and metabolite levels were measured with capillary electrophoresis mass spectrometry (CE-MS). Heatmap identifies metabolites with the highest fold change after coculture. FH, fumarate hydratase; SDHB, succinate dehydrogenase B; MDH2, malate dehydrogenase 2; NDUFA11, NADH:Ubiquinone Oxidoreductase subunit A11; COX4I2, cytochrome c oxidase subunit 4 isoform 2; ATP5C1, ATP synthase F1 subunit gamma; FBP1, fructose-bisphosphatase 1; PGLS, 6-phosphogluconolactonase; RPE, ribulose-5-phosphate-3-epimerase.

To determine whether tumor growth and survival within bone is associated with changes in metabolism in patients with prostate cancer, in silico analysis was performed with transcriptome data from prostate cancer bone metastases as compared to primary prostate tumors using the Kumar *et al.* 2016 dataset of laser-capture microdissected samples taken at rapid autopsy (*10*). This dataset is composed of transcriptome data on 22 primary and 154 metastatic tumors from multiple sites in 63 men with metastatic castrate-resistant prostate cancer (mCRPC) taken at rapid autopsy. The samples have been laser-capture microdissected, ensuring that the transcriptome data obtained are from the cancer cells and not the surrounding stroma. Using the Molecular Signatures Database Cancer Hallmark Gene Set, gene set enrichment analysis (GSEA) demonstrated that while only OXPHOS reached significance, reflecting the large number of genes examined and heterogeneous nature of the patient samples, the top five gene sets enriched in bone metastatic prostate cancer were associated with metabolism (Table 1). Using a metabolism-specific gene set analysis, six metabolic pathways were found to be significantly up-regulated in prostate cancer bone metastasis, as compared to primary prostate cancer, including OXPHOS, the TCA cycle, reactive oxygen species (ROS) detoxification, and glutathione metabolism (Table 2). Analysis of gene expression changes across different metastatic sites revealed bone-specific changes in metabolic pathways (Fig. 1, A to C, and fig. S1). Fifty-seven genes were identified as being part of the TCA cycle, of which 25 of 57 (44%) were significantly altered in bone metastases versus primary tumors, while 13 of 57 were altered in liver and 2 of 57 in lung metastases. Eighty-one genes were identified as being part of the electron transport chain, of which 23 of 81 (28%) were significantly altered in bone metastases versus primary tumors, while 1 of 81 was altered in liver and 11 of 81 in lung metastases. Nineteen genes were identified as being part of the PPP, of which 5 of 19 (26%) were significantly altered in bone metastases versus primary tumors, while 0 of 19 were altered in liver or lung metastases, suggesting that alterations in the PPP are specific to the bone microenvironment and not a generalized metabolic response to metastasis. The PPP was the seventh most up-regulated metabolic gene set in prostate cancer bone metastases (Table 2). Despite not quite reaching significance [false discovery rate (FDR) = 0.26] in GSEA analysis, the enrichment and specificity of PPP dysregulation for bone metastasis (Fig. 1, C and D) suggest a dependence on the bone microenvironment for PPP activity in prostate cancer bone metastases.

**Table 1.** In silico GSEA of metabolic changes in metastatic sites compared to primary tumors from the Kumar dataset using GSEA cancer hallmark gene set. The top five gene sets up-regulated in prostate bone cancer samples compared to primary prostate tumors are listed. FDR, false discovery rate. ES, enrichment score; NES, normalized enrichment score; NOM, nominal; FWER, familywise error rate.

**Name**	**Size**	**ES**	**NES**	**NOM *P* value**	**FDR *q* value**	**FWER *P* value**
Oxidative phosphorylation	197	0.52	1.77	0.014	0.197	0.08
Adipogenesis	190	0.44	1.56	0.012	0.554	0.369
Reactive oxygen species	47	0.44	1.46	0.053	0.762	0.58
PI3K AKT MTOR signaling	103	0.34	1.42	0.04	0.718	0.642
Fatty acid metabolism	154	0.42	1.34	0.137	0.893	0.764

**Table 2.** In silico GSEA of metabolic changes in metastatic sites compared to primary tumors from the Kumar dataset using a metabolic specific gene set. The top seven up-regulated gene sets in prostate bone cancer samples compared to primary prostate tumors are listed. The top six were significant with a nominal *P* value < 0.05 and FDR *q* value < 0.25. ES, enrichment score; NES, normalized enrichment score; NOM, nominal, FDR; false discovery rate; FWER, familywise error rate.

**Name**	**Size**	**ES**	**NES**	**NOM *P* value**	**FDR *q* value**	**FWER *P* value**
Oxidative phosphorylation	81	0.6155	2.5274	0.0000	0.0000	0.0000
Citric acid cycle	57	0.4998	1.9323	0.0000	0.0088	0.0140
Glutathione metabolism	23	0.5627	1.7520	0.0040	0.0405	0.0950
Nucleotide metabolism	26	0.5203	1.6768	0.0000	0.0666	0.2000
ROS detoxification	31	0.4788	1.6068	0.0176	0.0945	0.3160
Fatty acid biosynthesis	22	0.5073	1.5612	0.0224	0.1114	0.4220
Pentose phosphate pathway	19	0.4631	1.4088	0.0805	0.2645	0.7810

To further investigate whether the bone microenvironment can induce metabolic changes within prostate cancer cells, we undertook metabolomic profiling of PC3 prostate cancer cells following coculture with a range of human and murine bone marrow stromal cells. Alterations in the levels of multiple metabolites were identified after coculture, with differences also seen between different bone marrow stromal cell lines (Fig. 1E and table S1). In keeping with our in silico analysis, metabolites of the PPP were elevated by coculture with all bone marrow stromal cell lines, with associated increased levels of NADPH [reduced form of nicotinamide adenine dinucleotide phosphate (NADP^+^)] and decreased levels of NADP suggesting increased flux through the PPP. Together, our in silico analysis of patient data and tumor-bone metabolic profiling demonstrate significant metabolic alterations within the prostate cancer–bone microenvironment, including the PPP, and identify bone as a site for elevated PPP activity in prostate cancer cells.

### G6PD is overexpressed in bone metastatic prostate cancer

**Fig. 2.**
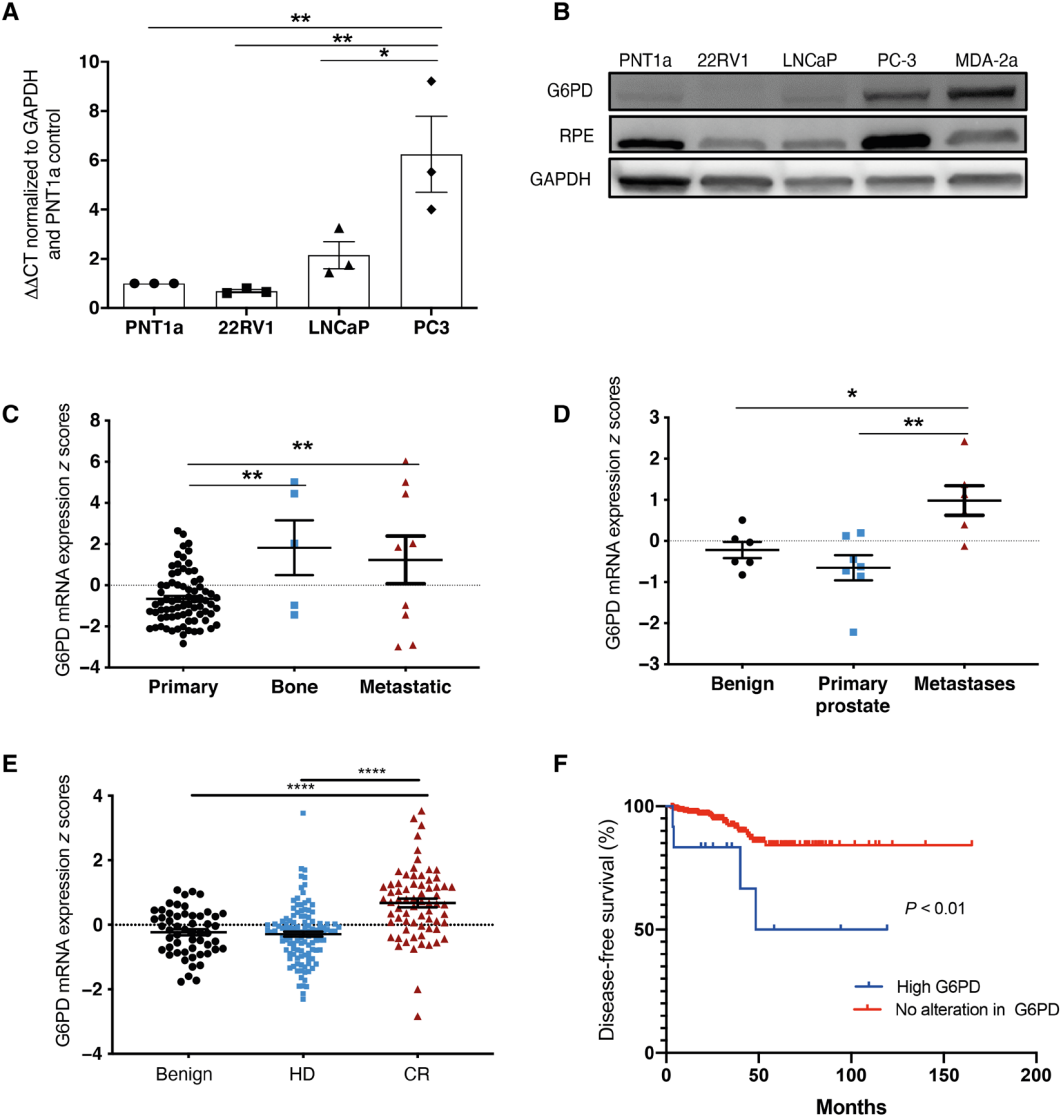
G6PD is overexpressed in bone metastatic prostate cancer. (**A**) mRNA expression of G6PD in different prostate cell lines. Statistical test used: one-way ANOVA with post hoc Tukey’s test. *P < 0.05 and **P < 0.01. Error bars = SEM. (**B**) Protein expression of PPP enzymes from benign (PNT1a), non–bone metastatic (22RV1, LNCaP), and bone metastatic prostate cancer cells lines [PC3, MDA PCA 2a (MDA-2a)]. (**C**) mRNA expression *z* scores of G6PD in patient samples from the Taylor 2010 dataset. Statistical test used: one-way ANOVA with Tukey’s post hoc test. (**D**) mRNA expression *z* scores of G6PD in patient samples from the Varambally 2005 dataset. Statistical test used: one-way ANOVA with Tukey’s post hoc test. (**E**) mRNA expression *z* scores of G6PD in patient samples from the Grasso 2012 dataset. HD, hormone dependent; CR, castrate resistant. Statistical test used: one-way ANOVA with Tukey’s post hoc test. (**F**) Progression-free survival curve in patient samples from the TCGA PanCancer Atlas dataset. Expression of G6PD was stratified into high expression (>2 SD from mean) and no alteration (<2 SD from mean). Statistical test used: Log-rank (Mantel-Cox) test. *P < 0.05, **P < 0.01, and ****P < 0.0001. Error bars = SEM.

Our transcriptomic and metabolomic analyses have converged to implicate the PPP in prostate cancer bone metastases. G6PD is the first and rate-limiting enzyme of the PPP, playing a critical role in both nucleotide precursor generation and protection of cells from oxidative damage. We evaluated G6PD gene and protein expression in human prostate cancer cell lines and found that G6PD expression was low in benign (PNT1a) and non–bone metastatic cell lines (22RV1 and LNCaP) and raised in bone metastatic cell lines (PC3, MDA PCa 2a, and DU145) (Fig. 2, A and B, and fig. S1), although interestingly, expression was low in C42B cells, a bone metastatic subline of LNCaP cells generated by isolation of tumor cells from bone following inoculation into castrated male mice (*11*). In contrast, the expression of TCA and OXPHOS enzymes was increased in cancer cells compared to benign cells but did not differ between bone metastatic and non–bone metastatic cell lines (fig. S3, A and B). To determine the clinical relevance of this, in silico analyses of multiple patient datasets were performed, with results from three distinct datasets demonstrating elevated G6PD in prostate cancer metastasis. The Taylor 2010 dataset is composed of 181 primary and 37 metastatic tumor samples from patients treated with radical prostatectomy (*12*). Transcriptomics was performed on tissue containing >70% tumor content on histological examination. Our analysis found that G6PD was significantly up-regulated in metastatic, including bone metastatic, prostate cancer samples compared to primary tumors (Fig. 2C). The Varambally 2005 dataset is a small dataset made up of 13 individual and 6 pooled samples from benign, primary, and metastatic prostate cancer tissue (*13*). Our analysis showed that G6PD gene expression was significantly up-regulated in metastatic samples compared to both benign and localized prostate cancer samples (Fig. 2D). The Grasso 2012 dataset is composed of benign, treatment-naïve high-grade localized, and heavily pretreated metastatic castrate-resistant patient samples (*14*). Our analysis showed that G6PD expression was significantly up-regulated as prostate cancer progressed from treatment-naïve to castrate-resistant (Fig. 2E), suggesting that G6PD may be up-regulated in response to disease progression and/or treatment. In support of this, analysis of the TCGA PanCancer Atlas dataset, a very large dataset with transcriptomics on 494 primary tumor samples from 494 patients (*15*), revealed a significant association between high G6PD expression and a reduction in disease-free survival. The Kaplan-Meier plot has not been adjusted for clinical prognostic factors or risk factors; therefore, it remains to be determined whether this reduction in disease-free survival is dependent on G6PD or whether G6PD is acting as a surrogate marker of Gleason score, prostate-specific antigen (PSA), or tumor category indicative of more advanced and aggressive disease (Fig. 2F). Together, our in silico analysis reveals that high levels of expression of G6PD, the rate-limiting enzyme of the PPP, are associated with disease progression, metastasis, and reduced progression-free survival.

### The bone microenvironment drives up-regulation of G6PD via IL-6

**Fig. 3.**
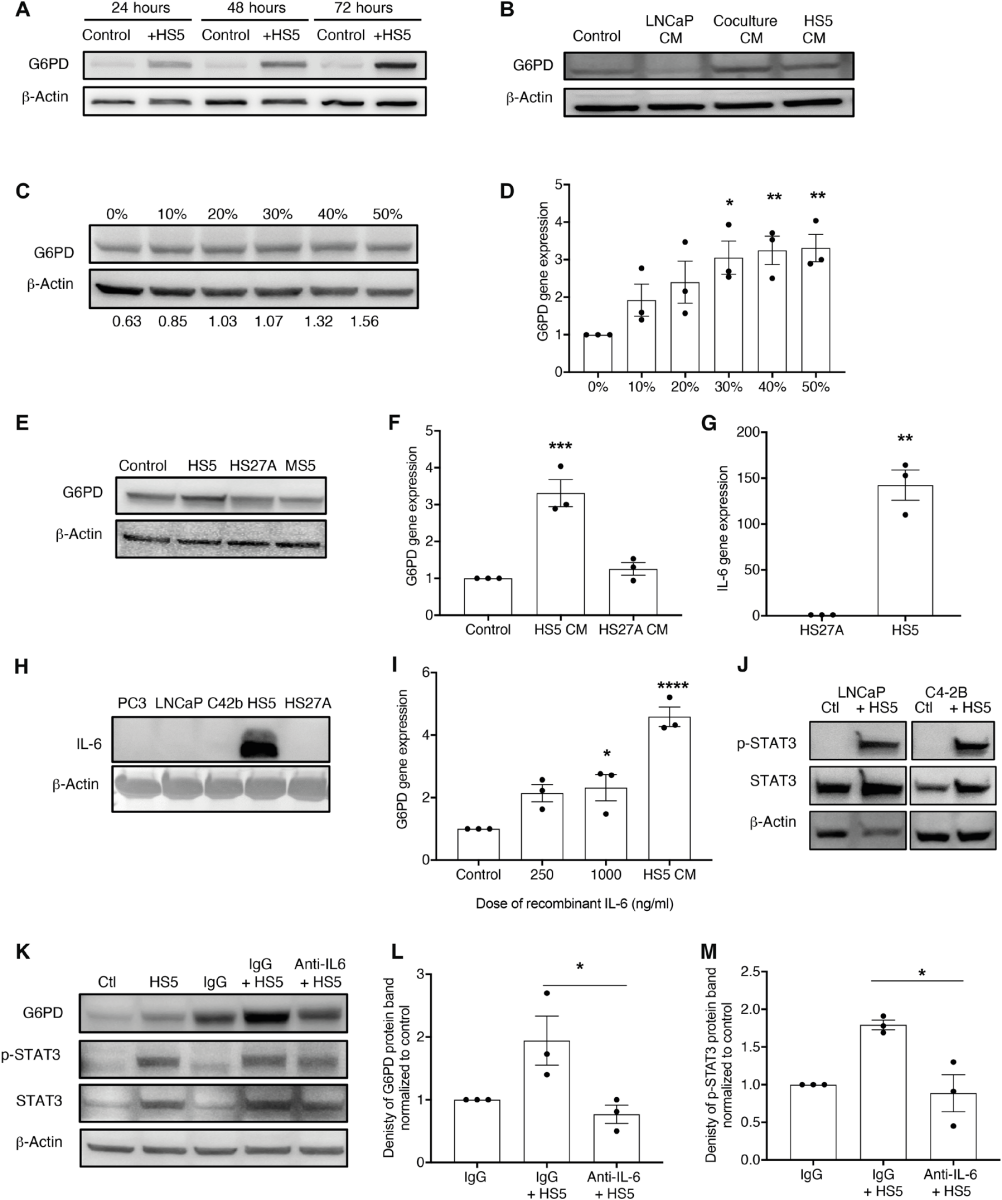
The bone microenvironment increases G6PD expression in prostate cancer cells via IL-6. (**A**) G6PD protein expression in LNCaP cells after transwell coculture with HS5 bone marrow stromal cells. (**B**) Effect of treatment of LNCaP cells with control (RPMI), 50% LNCaP CM, 50% coculture CM, or 50% HS5 CM on G6PD protein expression after 72 hours. (**C**) Increasing the proportion of HS5 CM increases G6PD protein expression in C4-2B cells at 72 hours. Densitometry quantification of G6PD band normalized to β-actin. (**D**) LNCaP cells treated with an increasing proportion of HS5 CM for 72 hours. G6PD mRNA levels examined by quantitative polymerase chain reaction (qPCR; normalized to Polr2a and control). (**E**) LNCaP cells treated with fresh RPMI or 50% CM from HS5, HS27A, or MS5 bone marrow stromal cells for 72 hours. (**F**) mRNA expression of G6PD normalized to Polr2a after 72-hour treatment with 50% HS5 or 50% HS27A CM. (**G**) qPCR of human IL-6 levels in HS5 and HS27A cells normalized to glyceraldehyde-3-phosphate dehydrogenase (GAPDH). (**H**) IL-6 expression in CM from prostate cancer cells (PC3, LNCaP, and C4-2B) and human stromal cell lines (HS5, HS27A). (**I**) LNCaP cells were treated for 72 hours with complete RPMI (Control), IL-6 (250 ng/ml), IL-6 (1000 ng/ml), or 50% HS5 CM and G6PD mRNA expression analyzed by qPCR (normalized to Polr2a). (**J**) HS5 CM (50%) activates p-STAT3 signaling in LNCaP and C4-2B cells. (**K**) LNCaP cells treated with 50% HS5 CM ± immunoglobulin G (IgG; 10 μg/ml) ± IL-6 neutralizing antibody (anti–IL-6; 10 μg/ml) for 72 hours. (**L**) Densitometry of G6PD band of LNCaP treated with 50% HS5 CM ± IgG (10 μg/ml) ± IL-6 neutralizing antibody (anti–IL-6; 10 μg/ml) for 72 hours. (**M**) Densitometry of p-STAT3 band of LNCaP treated with 50% HS5 CM ± IgG (10 μg/ml) ± IL-6 neutralizing antibody (anti–IL-6; 10 μg/ml) for 72 hours. *P < 0.05, **P < 0.01, ***P < 0.001, and ****P < 0.0001 as compared to control. Data represent means ± SEM.

Our metabolomic and in silico analyses have identified the PPP and, more specifically, G6PD as elevated in the prostate cancer–bone microenvironment. To further investigate the increase in G6PD within the bone microenvironment, we took advantage of prostate cancer cell lines with low G6PD expression (LNCaP and C42B). Following the culture of non–bone metastatic prostate cancer cells (LNCaP) in the presence and absence of bone marrow stromal cells (HS5), we demonstrated a time-dependent increase in G6PD expression (Fig. 3A). In contrast, no change was observed in expression of OXPHOS complexes or TCA enzymes following coculture of LNCaP prostate cancer cells with bone marrow stromal cells (fig. S4, A and B). G6PD up-regulation occurred in hypoxic- and glucose-free conditions, suggesting that the effect is not dependent on glucose or oxygen concentrations and can be seen in more physiological conditions (fig. S4, C and D). The up-regulation of G6PD was greater in androgen-deprived conditions, suggesting that current treatment may further drive this effect (fig. S4E). The increase in G6PD expression was apparent when prostate cancer cells were cultured with media conditioned by either bone marrow stromal cells alone or cocultures of prostate cancer cells and bone marrow stromal cells (Fig. 3B), with conditioned media (CM) from bone marrow stromal cells inducing a dose-dependent increase in G6PD protein and gene expression (Fig. 3, C and D). Together, this suggests that G6PD is elevated in response to a soluble factor released from bone marrow stromal cells and is not dependent on cellular cross-talk.

To determine what factor(s) may be responsible for the induction of G6PD expression, we first compared HS5 bone marrow stromal cells with the closely related HS27a cells, which are well characterized and known to release low levels of growth factors in comparison to HS5 cells. In contrast to HS5, CM from HS27a cells had no effect on the level of G6PD expression in prostate cancer cells (Fig. 3, E and F). IL-6 has previously been shown to be the second most altered gene in HS5 cells as compared to HS27a (*16*) and has been found to drive G6PD expression in renal cell carcinoma (*17*). In support of previous studies, IL-6 was found to be strongly expressed in HS5 bone marrow stromal cells as compared to HS27a bone marrow stromal cells or prostate cancer cell lines (Fig. 3, G and H). Treatment of prostate cancer cells with recombinant IL-6 was found to increase G6PD expression (Fig. 3I) and treatment of prostate cancer cells [either LNCaP or a bone-metastatic derivative, C4-2B (*11*)] with HS5 CM increased phosphorylation of signal transducer and activator of transcription 3 (STAT3), a known downstream mediator of IL-6 (Fig. 3, J and K). A neutralizing antibody to IL-6 was found to significantly reduce G6PD expression and STAT3 phosphorylation in prostate cancer cells treated with HS5 CM, demonstrating that bone marrow stromal cell–derived IL-6 can drive up-regulation of G6PD in prostate cancer cells (Fig. 3, K to M). Bisphosphonates are widely used in the treatment of the bone disease associated with prostate cancer bone metastases and have recently been suggested to inhibit the PPP in bladder cancer cells (*18*). This suggests a potential for pharmacological regulation of G6PD; however, our studies found no direct effect of zoledronic acid on expression of G6PD in PC3 prostate cancer cells (fig. S4F).

### G6PD regulates bone metastatic prostate cancer growth and migration

**Fig. 4.**
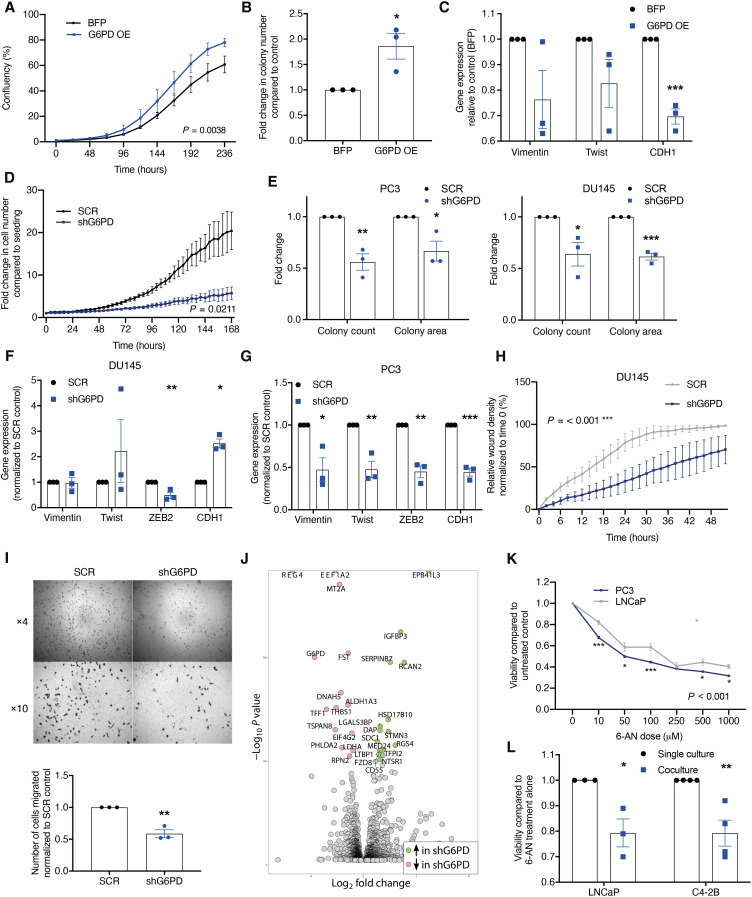
G6PD expression regulates bone metastatic prostate cancer growth and migration. (**A**) LNCaP control (BFP) or G6PD overexpressing (OE) cells, proliferation measured via IncuCyte system. Statistical test used: two-way ANOVA. (**B**) LNCaP BFP or G6PD OE cells’ colony formation. (**C**) LNCaP BFP or G6PD OE cells’ mRNA expression of EMT markers normalized to Polr2a. ***P < 0.001 as compared to BFP control. (**D**) Cell proliferation of DU145 measured via IncuCyte system. Statistical test used: two-way ANOVA. (**E**) Colony formation assay of SCR and shG6PD cells. *P < 0.05, **P < 0.01, and ***P < 0.001 as compared to SCR control. (**F** and **G**) mRNA expression of mesenchymal (Vimentin, Twist, and ZEB2) and epithelial (CDH1) markers SCR and shG6PD KD cells. Expression normalized to the housekeeping gene Polr2a and SCR control. *P < 0.05, **P < 0.01, and ***P < 0.001. (**H**) Relative scratch wound confluency. Statistical test used: two-way ANOVA. (**I**) Transwell migration assay at 24 hours. Migrated cell number normalized to SCR control. **P < 0.01. (**J**) RNA sequencing was performed on PC3-SCR control and G6PD knockdown. Volcano plot showing most differentially expressed genes (−log10*P*_adj_ > 100). (**K**) Cell viability after 24-hour treatment with 6-aminonicotinamide (6-AN). Statistical test used: two-way ANOVA with Sidak’s post hoc test. (**L**) Viability after treatment with 100 nM 6-AN for 72 hours in single culture or transwell coculture measured by Alamar Blue assay after the removal of the transwell normalized to untreated control. *P < 0.05 and **P < 0.01 as compared to single culture. Error bars = SEM.

**Fig. 5.**
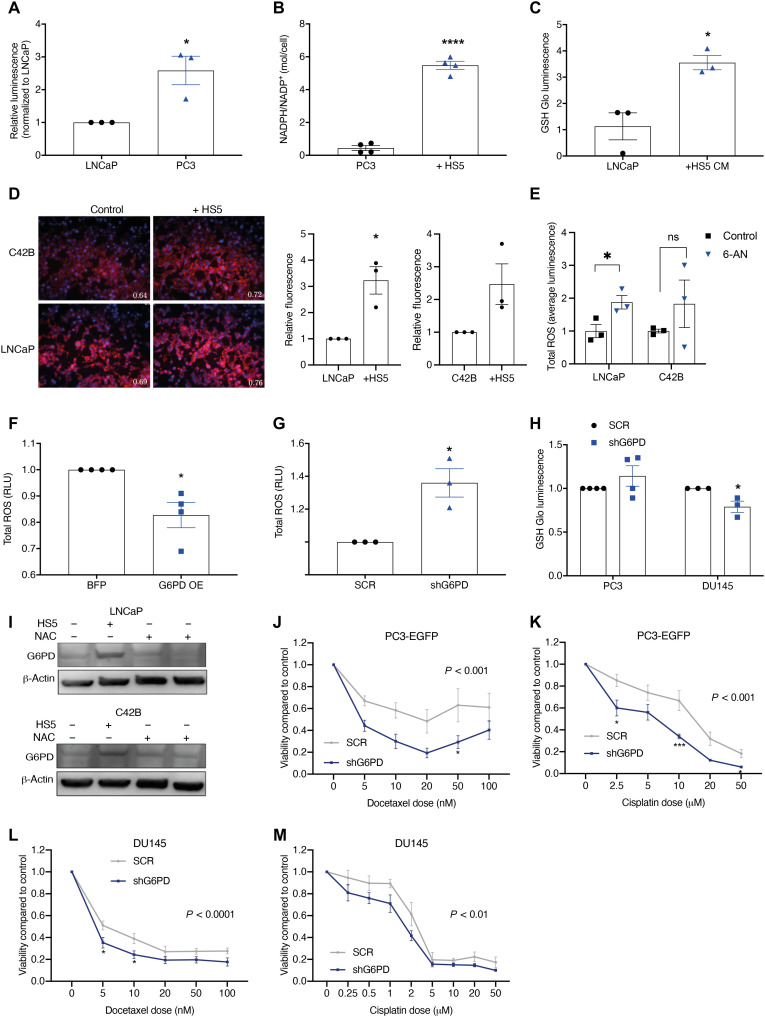
Bone marrow stromal cells and G6PD regulate the cellular redox state and chemosensitivity of bone metastatic prostate cancer cells. (**A**) GSH levels in the non–bone metastatic LNCaP cell line and the bone metastatic PC3 cell line. *P = < 0.05. (**B**) NADPH/NADP^+^ ratio in PC3 cells after 24-hour coculture with HS5 cells measured by CE-MS. ****P < 0.0001. (**C**) GSH levels in LNCaP cells after 72-hour treatment with HS5 CM, normalized to Alamar blue fluorescence. *P = < 0.05. (**D**) Mitochondrial ROS after 72 hours coculture with HS5 cells. Blue = Hoechst nuclear staining. Red = MitoSox mitochondrial ROS staining. Merged images taken on a Nikon Eclipse TE300 inverted microscope at 4× magnification. Images and values representative of three biological repeats. Quantified by MitoSox fluorescence reading and normalized to Hoechst fluorescence. *P = < 0.05. (**E**) Total ROS after 48-hour treatment with 100 nM 6-AN normalized to untreated control. *P = < 0.05. (**F**) Total ROS in G6PD OE cells normalized to BFP control *P = < 0.05. (**G**) Total ROS levels in PC3 SCR and shG6PD KD cells. (**H**) GSH levels in SCR and shG6PD KD cells. *P = < 0.05. (**I**) G6PD protein expression after 48-hour treatment with 50% HS5 CM ± 10 mM NAC. Blots representative of three biological repeats. (**J** to **M**) Cell viability in scrambled (SCR) and shG6PD KD (shG6PD) cells treated with docetaxel or cisplatin. Statistical test used for (J) to (M): two-way ANOVA with Sidak’s multiple comparisons post hoc test. *P < 0.05, ***P < 0.001 as compared to SCR. Error bars = SEM.

Having identified that the bone microenvironment drives G6PD overexpression, we explored the function of G6PD in prostate cancer cells. Overexpression studies were performed in low-G6PD expressing cells, with G6PD overexpressed in LNCaP cells, generating a stable cell line with a significant increase in gene and protein expression (fig. S5, A and B). Overexpression of G6PD resulted in a significant increase in prostate cancer proliferation and colony formation (Fig. 4, A and B), with a significant reduction in E-cadherin indicating a more mesenchymal phenotype (Fig. 4C). To further investigate the functional role of constitutive G6PD expression, G6PD was stably knocked down in PC3–enhanced green fluorescent protein (EGFP) and DU145 cells that are bone metastatic and exhibit high basal expression levels of G6PD, with a near-complete loss of gene and protein expression following knockdown (fig. S5, C to E). Knockdown of G6PD resulted in a significant reduction in proliferation and colony formation (Fig. 4, D and E, and fig. S5F) in both cell lines. A change in morphology in DU145 and PC3 cells was observed, with loss of G6PD expression resulting in smaller, more rounded cells indicative of a shift toward an epithelial phenotype (fig. 5, G and H). While not all EMT marker genes were indicative of epithelial transition, likely reflecting a spectrum of EMT plasticity, this was supported by reduced expression of mesenchymal markers (vimentin, Twist, and Zeb2) in DU145 and PC3 cells and elevated E-cadherin (CDH1) in DU145 cells (Fig. 4, F and G). In keeping with a more epithelial phenotype, knockdown of G6PD resulted in a significant reduction in migration using either a scratch-wound assay in DU145 cells or a modified Boyden chamber in PC3 and DU145 cells (Fig. 4, H and I, and fig. S5I). No significant difference in wound density was observed in PC3 cells with G6PD knockdown, perhaps reflecting the high rate of proliferation and resulting wound closure in PC3 cells (fig. S5J). Transcriptomic analysis of G6PD knockdown cells revealed the down-regulation of a number of genes implicated in prostate cancer cell proliferation and/or invasion, including LDHA, MT2A, and REG4 and up-regulation of genes involved in motility and cell death, including EPB41L3 and DAP (Fig. 4J and fig. S6, A and B). Pharmacological inhibition of G6PD activity using 6-aminonicotinamide (6-AN) resulted in a dose-dependent reduction in prostate cancer cell viability, which was greater when prostate cancer cells were cultured in the presence of bone marrow stromal cells, likely reflecting the elevated G6PD expression (Fig. 4, K and L, and fig. S7).

### Bone marrow stromal cells and G6PD regulate the cellular redox state and chemotherapy response of bone metastatic prostate cancer cells

In view of the known role of the PPP in cellular redox balance and the importance of ROS generation in chemotherapeutic response, we investigated whether targeting G6PD in bone metastatic prostate cancer cells could influence redox homeostasis and chemotherapy response. Bone metastatic PC3 prostate cancer cells were found to have higher levels of the antioxidant glutathione (GSH) when compared to non–bone metastatic LNCaP cells, along with higher G6PD (Figs. 2, A and B, and 5A). Coculture of PC3 or LNCaP prostate cancer cells with HS5 bone marrow stromal cells or CM was found to increase their antioxidant ability, with an increase in NADPH/NADP^+^ and GSH, respectively (Fig. 5, B and C). CM from HS5 bone marrow stromal cells was also found to increase ROS in LNCaP prostate cancer cells, with an increase in mitochondrial ROS (Fig. 5D). Pharmacological blockade of G6PD using 6-AN significantly elevated ROS in LNCaP prostate cancer cells (Fig. 5E). Similar trends toward an increase in ROS following treatment of C42B cells with HS5 CM or 6-AN were also observed (Fig. 5, D and E). Overexpression of G6PD was found to significantly reduce total ROS, whereas knockdown of G6PD was found to increase total ROS and reduce antioxidant levels, demonstrating a role for the PPP in controlling basal oxidative stress levels (Fig. 5, F to H). Prostate cancer cells were cultured with CM from HS5 bone marrow stromal cells and treated with the antioxidant *N*-acetylcysteine (NAC), resulting in a loss of the elevated G6PD expression in response to bone marrow stromal cells and suggesting that the increase in ROS could be further driving the elevated G6PD (Fig. 5I).

We show that pharmacological, genetic, and microenvironmental regulation of G6PD can alter the redox state of prostate cancer cells. Chemotherapeutic agents are known to generate ROS, leading to oxidative stress and apoptosis induction, with alterations in ROS levels shown to modulate sensitivity to chemotherapy. We therefore investigated whether alterations in G6PD can sensitize prostate cancer cells to chemotherapy. Docetaxel, cabazitaxel, and cisplatin all caused a significant increase in mitochondrial ROS (fig. S8, A and B). shG6PD enhanced the reduction in cellular viability caused by cisplatin and docetaxel in PC3-EGFP and DU145 cells (Fig. 5, J to M), indicating that G6PD blockade can increase the antitumor response to chemotherapy.

### G6PD knockdown inhibits prostate cancer growth in bone in vivo

**Fig. 6.**
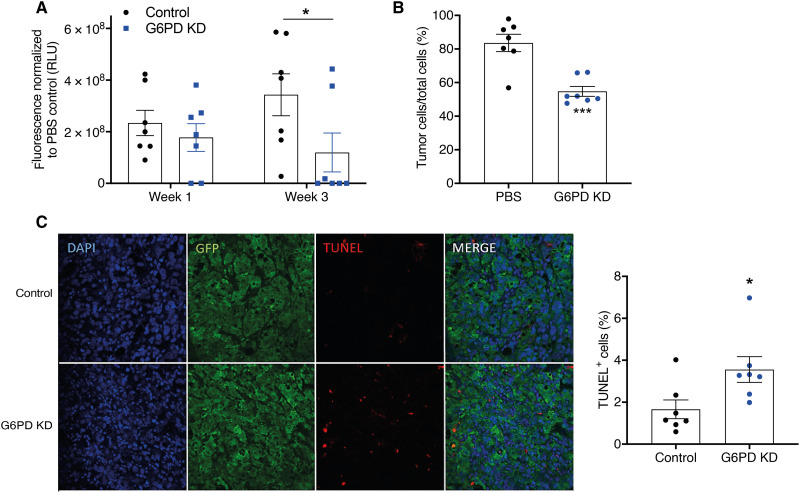
G6PD knockdown inhibits prostate cancer growth in bone in vivo. A total of 1 × 10^5^ PC3-EGFP SCR or shG6PD cells were inoculated into the tibias of 5-week-old SCID mice and tumor burden was monitored by fluorescence at the proximal tibia over time (*n* = 7). (**A**) Fluorescence values as measured in vivo by the IVIS imager at weeks 1 and 3. Statistical test used: one-way ANOVA with Tukey post hoc test. *P = < 0.05. (**B**) Tumor burden within bone was quantitated by immunohistochemistry and histomorphometry, with tumor cells visualized by GFP expression. Statistical test used: Mann-Whitney *U* test. ***P < 0.001. (**C**) Apoptotic tumor cells were identified by dual positivity for GFP and TUNEL. Statistical test used: Mann-Whitney *U* test. *P < 0.05. Error bars = SEM.

Our data suggest that G6PD expression is elevated in the bone microenvironment and plays a key role in prostate cancer cell growth; however, the function of G6PD in prostate cancer growth within the bone microenvironment in vivo is unknown. We therefore used a xenograft model in nude mice to investigate whether targeting G6PD inhibits prostate cancer tumor growth in vivo. PC3-EGFP G6PD knockdown cells or a scrambled control were injected via intratibial inoculation into the right leg of nude mice and longitudinal tumor growth was assessed by in vivo fluorescence imaging. Mice were inoculated with the same number of viable cells (2 × 10^5^), with no major difference in viability observed immediately after inoculation (SCR control 85% and G6PD KD 95%). A significant reduction in tumor burden was detected in mice bearing PC3-shG6PD tumors as compared to control PC3 tumors, as detected by in vivo fluorescence imaging and ex vivo immunohistochemistry (Fig. 6, A and B). Quantitation of apoptotic tumor cells by TUNEL (terminal deoxynucleotidyl transferase–mediated deoxyuridine triphosphate nick end labeling) staining revealed a significant increase in tumor cell apoptosis in PC3-shG6PD tumors as compared to mice bearing PC3 control cells (Fig. 6C). Overall, the loss of G6PD expression in prostate cancer cells resulted in a reduction in tumor growth and survival within bone.

## DISCUSSION

Increasingly it is being recognized that stromal cells can influence the metabolism and behavior of cancer cells (*19*–*21*). This study has explored the metabolic alterations induced by the bone microenvironment in patient samples and prostate cancer cell lines, with a focus on the cellular interactions between prostate cancer cells and bone marrow stromal cells. We demonstrate that the PPP and in particular its rate-limiting enzyme G6PD are up-regulated in prostate cancer bone metastasis. We provide evidence for a specific role of the bone microenvironment to increase G6PD expression and demonstrate that bone marrow stromal cells can drive increased G6PD expression via secretion of IL-6. This bone marrow stromal cell–G6PD axis regulates the redox state of prostate cancer cells, with a loss of G6PD elevating ROS, decreasing antioxidants, and enhancing the chemosensitivity of prostate cancer cells. G6PD levels control prostate cancer cell growth both in vitro and in vivo, with G6PD inhibition, either by genetic modulation or pharmacological inhibition, resulting in a reduction in growth and survival. By combining analysis of patient data, metabolomics, in vitro mechanism, and preclinical models, we have revealed a previously unknown mechanism by which the bone microenvironment induces metabolic perturbations to promote advanced prostate cancer.

Metabolic changes in cancer cells are one of the key drivers of disease progression (*22*), yet their role in advanced cancer, and particularly bone metastasis, is poorly understood. Insight from metabolomic profiling of patients with advanced prostate cancer is limited, with most studies using primary prostate tumor biopsies, reflecting the challenges in collection and analysis of samples from prostate cancer bone metastases. Thysell *et al.* (*23*) used gas chromatography–time-of-flight mass spectrometry to identify metabolic changes in prostate cancer bone metastases as compared to primary prostate or normal adjacent bone, including an increase in cholesterol. While this study did not discern changes in the pathways identified in the current study, including OXPHOS, TCA, and the PPP, this likely reflects the different mass spectrometry separation techniques used and focus of the analysis, along with the low number of patient samples and heterogeneous disease stage. The significance of metabolic dysfunction in advanced prostate cancer is exemplified by our in silico analysis, using a powerful dataset in which tumor cells were isolated by laser-capture microdissection; thus, removing potential contamination from host cells and revealing metabolic pathways are among the most altered of all the cancer hallmark pathways in metastases as compared to primary tumors. When comparing specific metastatic sites, changes in the OXPHOS, TCA, and PPP pathways were much more prominent in bone metastases, with the greatest bone specificity observed in the PPP.

Complementary to our in silico analysis, metabolomic profiling of prostate cancer cells following culture with distinct bone marrow stromal cell lines revealed multiple metabolic changes, with a consistent elevation in glycolysis and the PPP induced by all stromal lines. This was associated with a significant increase in NADPH and decrease in NADP^+^, supporting a role for increased PPP flux during the coculture. In vitro studies supported the in silico and metabolomic profiling, demonstrating increased expression of components of the PPP in bone metastatic prostate cancer cell lines and increased expression following culture with bone marrow stromal cells. Notably, the TCA and OXPHOS pathways were not similarly dysregulated, suggesting a specificity for elevation of the PPP in the prostate cancer–bone microenvironment.

The PPP branches from glycolysis at the first committed step of glucose metabolism and is required for synthesis of ribonucleotides and as a major source of NADPH. The rate-limiting enzyme of the PPP, G6PD, was first suggested as a biomarker for prostate cancer over 30 years ago (*24*, *25*), with increased G6PD activity noted in patients with prostate cancer as compared to benign prostatic hyperplasia (BPH). More recently, G6PD levels have been found to be elevated in prostate cancer, with G6PD proposed as a mediator of AR signaling (*26*). To date, while the PPP is attracting increasing attention in the cancer field, with studies in lung (*27*), colorectal (*28*), gastric (*29*), kidney (*30*), and breast (*31*) cancer demonstrating G6PD expression associated with advanced disease and metastasis, the contributions of the PPP to the final stage of bone metastases remain unexplored. Here, we provide evidence that the PPP and in particular G6PD are up-regulated in bone metastatic prostate cancer patient samples, with expression correlating with disease severity as characterized by hormone sensitivity and disease-free survival. This relationship was seen in multiple datasets with large and small patient numbers strengthening the evidence for this effect.

Up-regulation of G6PD could be recreated in an in vitro model by coculturing LNCaP cells with HS5 bone marrow stromal cells, supporting a role for the bone microenvironment driving this effect and providing evidence of stromal regulation of the PPP. Up-regulation of G6PD was not affected by altering the glucose concentration of the media, or by performing coculture under hypoxic conditions, suggesting that this effect is likely to be seen in more physiological conditions. There was an overall decrease in G6PD under hypoxic conditions, which contrasts with what has been shown by others in different cell types. Chettimada *et al.* (*32*) showed that G6PD expression was increased by hypoxia in pulmonary artery smooth muscle cells, while Gao *et al.* (*33*) showed that hypoxia caused an increase in G6PD expression in PC12 rat pheochromocytoma cells, a classic O_2_-sensitive excitable cell, but not in nonexcitable Buffalo rat liver cells. It has been suggested that it is the ROS generated under hypoxic conditions that promote G6PD expression via an oxidative stress-sensitive transcription factor, rather than hypoxia itself. Indeed, Nrf2 could be such a transcription factor, responding to ROS and activating PPP genes (*34*, *35*). Therefore, how G6PD expression is regulated by low oxygen tension is still not clear and remains controversial.

Up-regulation of G6PD by bone marrow stromal cells was found to be driven, at least in part, by stromal-derived IL-6, with blockade of IL-6 using a neutralizing antibody preventing the elevated G6PD. In line with this, activation of STAT3, the downstream mediator of IL-6 signal transduction, was elevated by coculture of prostate cancer cells with bone marrow stromal cells, and subsequently reduced following IL-6 blockade. It has long been recognized that IL-6 promotes tumor growth and survival (*36*), with elevated levels of circulating IL-6 detected in patients with untreated mCRPC or CRPC (*37*, *38*), and serum levels correlated with shorter survival time (*39*). Targeting IL-6 in the clinic has yielded mixed results with a range of toxicities (*40*–*42*), and it is possible that targeting G6PD as a downstream mediator may represent a more refined approach.

Up-regulation of G6PD protein was enhanced in androgen-deprived conditions. Most men with metastatic prostate cancer are treated with ADT as first-line therapy, and therefore, these data indicate that current treatments may in fact be enhancing the G6PD up-regulation seen. This would be in keeping with our in silico analysis demonstrating that G6PD gene expression increases as patients progress from treatment-naive hormone-sensitive disease to heavily treated castrate-resistant disease. However, this is in contrast with work by Tsouko *et al.* (*26*) that showed that G6PD protein levels are increased with androgen treatment. Interestingly they found that knockdown of the androgen receptor led to decreased G6PD protein levels. The endogenous steroid hormone dehydroepiandrosterone (DHEA) is produced by the adrenal glands as a metabolic precursor of androgen and estrogen and is a known uncompetitive inhibitor of G6PD (*43*). DHEA is the most abundant steroid in humans and an important source of androgens. When metabolized by prostate cells, DHEA can contribute up to one-sixth of dihydrotestosterone present in the prostate (*44*). In prostate cancer rat models, DHEA has been shown to confer protection against prostate cancer (*45*), while in patients, low serum levels of DHEA have been shown to correlate with high Gleason score, advanced clinical stage, and poor prognosis (*46*) in hormone-naïve prostate cancer (*47*), and worse cancer-specific survival among patients with mCRPC (*48*). These studies, however, have not correlated DHEA levels with G6PD expression or PPP activity. The role of DHEA in prostate cancer progression remains controversial as DHEA has also been shown to stimulate prostate cancer proliferation and PSA expression in prostate cancer models in vitro and in vivo (*49*, *50*) via PI3K/Akt signaling. The role of androgens in modulating G6PD expression and activity therefore remains far from clear, and further work needs to be done to fully elucidate the feedback mechanisms that may be involved between the androgen signaling and the PPP pathway.

Supporting a role for G6PD in bone metastasis, manipulation of G6PD expression, or activity was found to directly affect prostate cancer growth and metastatic behavior, with knockdown of G6PD resulting in a reduction in prostate cancer proliferation, colony formation, mesenchymal markers, and migration. The precise mechanisms responsible for the functional effect of G6PD remain to be determined, with increasing evidence to support metabolic control of EMT (*51*). RNA sequencing (RNA-seq) confirmed gene changes in prostate cancer cells with knockdown of G6PD that suggested a more epithelial, less invasive, and proliferative phenotype. While the mechanism underlying the transcriptomic changes following G6PD knockdown is unknown, it is intriguing to speculate whether G6PD may act like a transcription factor. In vivo, knockdown or overexpression of G6PD has previously been shown to affect tumor growth in subcutaneous xenograft models in a small number of other cancer systems (*17*, *28*, *52*, *53*), highlighting the importance of this pathway in cancer cell survival, but not incorporating key components of the tumor microenvironment. In prostate cancer, while increased G6PD expression has been observed in a murine model of prostate cancer (*26*), the function of G6PD either in the primary tumor or in a metastatic setting in vivo remains unclear. In the current study, we use a well-characterized model of prostate cancer bone disease, demonstrating that knockdown of G6PD expression in prostate cancer cells resulted in a significant reduction in tumor burden within bone in vivo, associated with an increase in tumor cell apoptosis. It is intriguing to speculate whether overexpression of G6PD in nonmetastatic prostate cancer would be sufficient to drive bone metastasis, a challenging experiment due to the lack of well-characterized in vivo models in which prostate cancer spreads from the primary tumor site to the bone. The current study highlights the potential for targeting the PPP in the treatment of prostate cancer bone metastasis. Currently, pharmacological targeting of the PPP in vivo is challenging because of a lack of specific inhibitors. In vitro, pharmacological inhibition of G6PD using 6-AN was found to significantly reduce proliferation; notably, the reduction in prostate cancer cell viability was greater when cells were cultured in the presence of bone marrow stromal cells. This suggests that pharmacological inhibition of G6PD is still effective in the prostate cancer–bone microenvironment and can overcome the increase in G6PD expression induced by the bone microenvironment, providing further support for targeting the PPP in prostate cancer bone metastasis.

The PPP and G6PD play an important role in modulating the redox state of the cell, and it was found that knockdown of G6PD led to increased ROS levels and decreased GSH, while G6PD overexpression resulted in decreased ROS. These results suggest that knockdown of G6PD leads to reduced NADPH levels, leading to increased sensitivity to oxidative stress, while G6PD overexpression increases NADPH, with greater protection against ROS. In keeping with this, it was found that knockdown of G6PD could sensitize prostate cancer cells to cisplatin and docetaxel chemotherapy. These results are supported by work performed in other cancer models, where alterations in G6PD expression have been associated with changes in NADPH/NADP^+^ ratios and cisplatin sensitivity (*28*, *54*, *55*). In addition to direct regulation of the redox state by G6PD, we also demonstrated that bone marrow stromal cells increase ROS and the antioxidant ability of the cells. Collectively, our data suggest that targeting G6PD in combination with currently used chemotherapies may improve their efficacy, due to alterations in the cellular redox state. This combined approach is particularly attractive in the setting of prostate cancer bone metastasis, where bone marrow stromal cells induce a similar alteration in redox state and which is strongly associated with drug resistance.

G6PD deficiency is one of the most common inherited enzyme defects worldwide and occurs almost exclusively in males. This inborn error of metabolism predisposes those affected to red blood cell breakdown. There is increasing evidence that this deficiency may offer protection against carcinogenesis, with a recent study revealing a reduced susceptibility to cancers of endodermal origin (stomach, colon, and liver) in carriers of G6PD deficiency (*56*). In addition, those with a G6PD deficiency are thought to have some protection against malaria (*57*), increasing the interest in developing an effective pharmacological inhibitor of G6PD for clinical use.

Once malignancies such as prostate cancer colonize bone, treatment is largely palliative and the disease becomes incurable. The complex relationship between tumor cells and the bone microenvironment represents the key driver of disease progression; hence, identifying new mechanisms to disrupt this symbiotic relationship is key to identifying effective therapeutic approaches. With the recent recognition of the importance of metabolic reprogramming in cancer progression, our study reveals a metabolic mechanism by which the bone microenvironment supports prostate cancer growth and survival, by up-regulation of G6PD, the rate-limiting enzyme of the PPP. Furthermore, we show how elevated G6PD contributes to prostate cancer growth and survival within this unique tumor microenvironment. While specific and selective pharmacological blockade of the PPP in vivo remains challenging, we provide evidence to demonstrate the antitumor effect of G6PD blockade in vivo in prostate cancer bone metastasis, supporting the growing interest in targeting metabolic reprogramming for the prevention and/or treatment of prostate and other cancers.

## MATERIALS AND METHODS

### Cell culture

PC3, PC3-EGFP, C4-2B, LNCaP, PNT1a, DU145, 22Rv1, HS5, HS27A, 2T3, and ST2 cells were routinely maintained in RPMI 1640 medium (Sigma-Aldrich, catalog no. R0883) supplemented with 10% fetal bovine serum (FBS; catalog no. FCS-SA, Labtech), 1% l-glutamine (catalog no. G7513, Sigma-Aldrich), 1% penicillin/streptomycin (catalog no. P0781, Sigma-Aldrich), 1% vitamins (catalog no. 11120-037, Gibco), 1% sodium pyruvate (S8636, Sigma-Aldrich), and 1% nonessential amino acids (catalog no. M7145, Sigma-Aldrich) (complete RPMI medium). By maintaining cells in the same culture medium, we avoid any metabolic response to differences under culture conditions. Cells were routinely validated by genotyping and periodically tested to ensure the absence of mycoplasma contamination. All cell lines were maintained at 37°C in a humidified 5% CO_2_ atmosphere. For transwell coculture, cells were seeded at a density of 5 × 10^5^ in six-well plates or transwell filters (0.4-μm pore size; catalog no. 3412, Corning). CM was obtained from 72 hours of single or prostate cancer–bone marrow stromal cell transwell coculture and centrifuged at 1500*g* for 5 min, and the supernatant was used fresh or stored at −20°C for later use. Unless otherwise specified, CM was used at a dilution of 1:1 in complete RPMI media. Recombinant IL-6, neutralizing antibody, and immunoglobulin G (IgG) isotype control were obtained from R&D Systems (206-IL; MAB2061; MAB004, R&D Systems). Docetaxel (catalog no. 01885, Sigma-Aldrich) and cabazitaxel (catalog no. C046500, Toronto Research Chemicals) were reconstituted in dimethyl sulfoxide (DMSO) to a concentration of 0.5 M and stored in single-use aliquots at −20°C until use. Cisplatin (catalog no. 479306, Sigma-Aldrich) was reconstituted to a 1 M solution in 0.9% NaCl and stored at 4°C. 6-AN (catalog no. A68203, Sigma-Aldrich) was reconstituted to 1 M in DMSO and stored at −20°C until use. Hypoxia studies were performed using a hypoxia chamber (New Brunswick, Galaxy 48R) set to 37°C, 0.5% O_2_. For glucose-free conditions, RPMI with no glucose (catalog no. R1383, Sigma-Aldrich) was used, while for androgen deprivation conditions, RPMI with 10% charcoal-stripped FBS (catalog no. A3382101, Gibco) was used, giving cells 72 hours to allow purging of androgens from the cells before treatment conditions were started.

Cell proliferation was measured using bright-field or GFP fluorescent images taken on an IncuCyte camera and analyzed using IncuCyte analysis software (Essen Biosciences, CI, USA). Cell viability was assessed using the Alamar Blue assay (catalog no. R7017, Sigma-Aldrich), read using a fluorometer (FLUOstar Omega, BMG labtech; excitation: 544 nm, emission 590 nm).

For colony formation, cells were seeded at 1 × 10^4^ in a six-well plate and left for 7 days and then stained with crystal violet fixing/staining solution [0.05% crystal violet, 1% formaldehyde, 1× phosphate-buffered saline (PBS), and 1% methanol]. Colonies were quantified using a Celigo imaging system (Nexcelom, MA, USA). Cell migration was analyzed using the IncuCyte WoundMaker Assay or via transwell migration through transwell inserts containing polycarbonate membranes with 8-μm pore size (catalog no. 3422, Corning).

### Capillary electrophoresis mass spectrometry analysis

Capillary electrophoresis mass spectrometry analysis was performed at the Institute for Advanced Biosciences, Keio University, Tsuruoka, Japan, under the supervision of T.S. Cells were washed twice with 5% mannitol solution and covered with 700 μl of methanol containing 25 μM internal standards [20 μM each of methionine sulfone, 2-(*N*-morpholino)-ethanesulfonic acid and d-camphor-10-sulfonic acid] for 10 min. The resulting extracts were mixed with 200 μl of Milli-Q water and 400 μl of chloroform and centrifuged at 4600*g* for 15 min at 4°C. Then, 400 μl of the aqueous phase of the sample solution was subjected to ultrafiltration through a 5-kDa cutoff filter (Human Metabolome Technologies, Tsuruoka, Japan) to remove proteins. The filtrate was centrifugally concentrated and dissolved in 25 μl of Milli-Q water that contained reference compounds (200 μM each of 3-aminopyrrolidine and trimesic acid) immediately before metabolome analysis. The concentrations of all the charged metabolites were measured by capillary electrophoresis time-of-flight mass spectrometry (Agilent Technologies, Palo Alto, CA, USA) using the methods described previously (*58*, *59*). Analysis was performed using MasterHands Software (v2.13.0.8h, Keio University).

### Immunoblot experiments

Complete cellular extracts were lysed using 40 to 100 μl of radioimmunoprecipitation assay buffer (catalog no. R0278, Sigma-Aldrich) with 4% protease inhibitor (catalog no. 11873580001, Roche) and 1% phosphatase inhibitor cocktail 2 (catalog no. P5726, Sigma-Aldrich) following the manufacturer’s recommendations.

Lysate concentration was determined used a Pierce bicinchoninic acid (BCA) assay kit (catalog no. 23225, Thermo Fisher Scientific). Known concentrations of protein were mixed with appropriate volumes of 4× LDS Sample buffer and 10× Sample Reducing Agent (Invitrogen, Carlsbad, USA) and denatured at 95°C for 5 min. An equal amount of protein (40 to 60 μg) was loaded into a 4 to 20% gradient SDS–polyacrylamide gel electrophoresis (Bio-Rad) and transferred to a polyvinylidene difluoride membrane (Bio-Rad) using a semidry transfer system (Trans-Blot Turbo, Bio-Rad). Membranes were exposed to the following antibodies: rabbit anti-G6PD (CST; 8866; 1:1000), rabbit anti-RPE (Abcam; ab98354; 1:1000), rabbit anti–glyceraldehyde-3-phosphate dehydrogenase (GAPDH) (CST; 5174; 1:1000), mouse anti–β-actin (Sigma-Aldrich A2228; 1:20,000), rabbit anti-IL-6 (Abcam; ab6672; 1:1000), rabbit anti-p-STAT3 (CST; 9145; 1:2000), rabbit anti-STAT3 (CST; 12640; 1:2000), mouse anti-OXPHOS complex kit (Life Technologies; 457999; 1:250), rabbit anti-SDHA (CST; 11998; 1:1000), rabbit anti-fumarase (CST; 4567; 1:1000), mouse anti-citrate synthase (Santa Cruz; sc-390693; 1:1000), rabbit anti-PSA (Abcam; ab53774; 1:1000) combined with secondary anti-rabbit (CST; 7074; 1:5000), or anti-mouse (CST; 7076; 1:5000) antibodies conjugated to horseradish peroxidase. Membranes were developed using Clarity Western ECL Substrate (Bio-Rad) and imaged on a gel doc system (Alliance 6.7, Uvitech, Cambridge, UK).

### RNA isolation

Total RNA was isolated from cultured cells using an RNeasy kit (catalog no. 74106, Qiagen) according to the manufacturer’s instructions. RNA was quantified using a NanoDrop 100 spectrophotometer (Thermo Fisher Scientific). Quality of the RNA was assessed by measuring the ratios of absorbance (*A*_260_/*A*_280_ and *A*_260_/*A*_230_); ratios greater than 1.8 were considered suitable for further analysis.

### cDNA synthesis

One microgram of RNA was deoxyribonuclease (DNase)–treated using a DNase kit (catalog no. DNASE-50, Primer Design) to eliminate any contaminating genomic DNA. This was then reverse-transcribed into cDNA using 20 μl of Nanoscript 2 (Cat. RT-premix-48, Primer Design) and cycled in a Veriti thermocycler (Applied Biosystems) according to the manufacturer’s instructions.

### Quantitative real-time polymerase chain reaction

cDNA was appropriately diluted with ribonuclease/DNase-free water and used for quantitative real-time polymerase chain reaction (qRT-PCR) with either Taqman or Fast SYBR Green reagents. Taqman qPCR was performed using Taqman primers (Life Technologies) and Taqman Universal Mastermix (catalog no. 4440041, Life Technologies) according to the manufacturer’s instructions. SYBR Green qPCR was performed using SYBR green or Qiagen primers and Fast SYBR Green Mastermix (Life Technologies) following the manufacturer’s instructions.

The expression of genes was normalized against that of a housekeeping gene, POLR2A (RNA polymerase II subunit A), when comparing different conditions in the same cell line, and GAPDH, when comparing between different cell lines. Plates were cycled on a Viia7 (Applied Biosystems) real-time thermal cycler according to the manufacturer’s instructions. Samples were plated with three technical repeats. Gene expression was analyzed by the ΔΔCT method in Excel (Microsoft Office).

### In silico analysis

The Kumar *et al.* 2016 dataset (*10*) was obtained via CBioPortal (*60*) (Center for Molecular Oncology, Memorial Sloan Kettering Cancer Center, USA). Taylor (*12*), Varambally (*13*), and Grasso (*14*) datasets were obtained via Gene Expression Omnibus (*61*) (National Center for Biotechnology Information, USA). Analysis was performed using Prism 6 (GraphPad) and GSEA (Broad Institute, USA). For individual gene sets, a one-way analysis of variance (ANOVA) was performed with post hoc Bonferroni test correction to adjust for multiple comparisons. GSEA was done using the GSEA cancer hallmark gene set or a curated metabolic specific gene set from Gaude and Frezza (*62*). Pathways were considered to be significantly altered in the GSEA analysis if they had a nominal *P* value < 0.05 and FDR *q* value < 0.25.

### Redox state quantification

MitoSOX Red (catalog no. M36008, Thermo Fisher Scientific) was used to measure mitochondrial superoxide. Total ROS levels were measured using a ROS-Glo Kit (catalog no. G8820, Promega) and glutathione levels were measured using a GSH Glo kit (catalog no. V6911, Promega, Wisconsin, USA).

### shRNA and ORF transfection

G6PD MISSION short hairpin RNA (shRNA; TRCN0000281204, TRCN0000281206, and TRCN0000281207), G6PD MISSION TRC3 open reading frame (ORF; TRCN000046117), and scrambled control (catalog no. SHC002) bacterial glycerol stocks and MISSION BFP TRC3 ORF (ORFBFPV) lentivirus were purchased from Sigma-Aldrich. Colonies were grown on agar with ampicillin (0.1 mg/ml). After 24 hours at 37°C, a single colony was picked and grown in LB broth with ampicillin (0.1 mg/ml) overnight on a shaker at 200 RPM. The plasmid was then purified using the Qiagen Endofree plasmid purification kit according to the manufacturer’s instructions. Plasmid DNA was eluted in water, and purity and concentration were checked using a Nanodrop 100 spectrophotometer.

Lentiviral packaging was performed in human embryonic kidney (HEK) 293T cells using a second-generation packaging system. Briefly, HEK 293T cells were seeded in 100-mm tissue culture dishes (Corning) and incubated at 37°C at 5% CO_2_ in a humidified incubator until they were ~90% confluent. Ten micrograms of transfer vector was added to 5 μg of psPAX2, 5 μg of pMD2.G, and 500 μl of Opti-MEM (catalog no. 31985062, Life Technologies). Separately, 30 μl of Lipofectamine 2000 (catalog no. 11668019, Life Technologies) was added to 500 μl of Opti-MEM. These were then mixed and incubated at room temperature for 20 min with regular inversion. The transfection solution (1 ml) was then added to the HEK 293T cells in 10 ml of antibiotic-free Dulbecco’s modified Eagle’s medium. Plates were incubated at 37°C for 72 hours, and culture supernatant containing the lentivirus was collected. Transfection was confirmed at this point by expression of fluorescent protein in the HEK 293T cells transfected with a control fluorescent construct. The supernatant was centrifuged at 4000 RPM for 10 min at 4°C and then filtered through a 0.45-mm polyethersulfone filter. The lentivirus was made into a 10 times concentrated stock using the Lenti-X Concentrator Solution (catalog no. 631232, Clontech). The solution was incubated at 4°C overnight before being centrifuged at 1500*g* for 45 min at 4°C. The supernatant was then removed, and the pellet was resuspended, flash-frozen in liquid nitrogen, and stored at −80°C.

Prostate cancer cells were seeded at 5 × 10^5^ in six-well plates, treated with 200 μl of virus and polybrene (8 μg/ml; catalog no. H9268, Sigma-Aldrich) to enhance transduction efficiency, and incubated for 72 hours at 37°C. Transduced cells were selected using puromycin (Sigma-Aldrich, catalog no. P7130) for 6 weeks (2 μg/ml for LNCaP and C4-2B; 4 μg/ml for PC3-EGFP and DU145). A puromycin kill curve of nontransfected LNCaP, C42B, and DU145 cells was performed over 14 days to confirm the appropriate dose of puromycin. Knockdown was confirmed by qPCR.

### RNA sequencing

RNA was DNase I–treated, cleaned, and concentrated according to the manufacturer’s instructions using Zymo RNA Clean and Concentrator-5 (R1013, Zymo). mRNA was enriched from total RNA according to the manufacturer’s instructions using a NEBnext poly(A) mRNA magnetic isolation module. First, mRNA was isolated and fragmented from total RNA using oligo dT beads and a magnetic rack. Beads were mixed with 1 μg of RNA and then washed twice. Samples were then heated to 65°C for 5 min before being washed. Tris buffer was added and samples were heated once again to 80°C and held at 25°C to perform the first elution of the mRNA from the beads. Beads were subsequently washed, and mRNA was eluted and fragmented with the addition of first strand synthesis reaction buffer and random primer mix. Samples were heated to 94°C for 15 min and held at 4°C. Sequencing libraries were prepared according to the manufacturer’s recommendations using the NEBNext Ultra II RNA Library Prep kit (E7770S, NEB). Briefly, first- and second-strand cDNA synthesis was performed using mRNA isolated as above. Double-strand cDNA was purified using NEBNext sample purification beads, and DNA was eluted in 0.1× tris-EDTA buffer. End prep reaction and adaptor ligation were performed, and purification was done using sample purification beads. RNA and DNA quantification was performed using HS RNA and HS DNA assay kits (Q32852 and Q32851, Invitrogen) and a Qubit fluorometer (Life Technologies) according to the manufacturer’s instructions. RNA and DNA quality was determined using High Sensitivity RNA Screentape and D1000 High Sensitivity DNA Screentape, respectively, on an Agilent 4200 tapestation system according to the manufacturer’s instructions.

Single-indexed and multiplexed samples were run on an Illumina Next Seq 500 sequencer using a NextSeq 500 v2 kit (150 cycles) (FC-404-2005) for paired-end sequencing (75 bases × 2). Results from RNA-seq were aligned to the human genome assembly version 38 (hg38 or GRCh38). At least 14 million aligned reads were obtained per sample.

For differential expression analysis, sequencing reads were demultiplexed based on the sample index and aligned to the human genome (hg38) using the STAR (Spliced Transcipts Alignment to a Reference) aligner (*63*). Differential expression analysis was performed using DESeq2 (*64*) between two groups designated as SCR (scrambled) and G6PD-KD (G6PD knockdown).

### In vivo model

Five-week-old male CB17 severe combined immunodeficient (SCID) mice were purchased from Charles Rivers (Wilmington, MA, USA) and allowed to acclimatize to their new environment for 1 week before the start of the experiment. All animals were maintained in conditions in accordance with the Animal (Scientific Procedures) Act 1986, and the guidelines of the University of Oxford. All procedures were approved by the Home Office under project license PCCCC8952. Animals were maintained in individually ventilated cages under controlled temperature (19° to 23°C) and light (12-hour light/dark cycles) with food and water ad libitum. Mice were anesthetized using 3% isoflurane for induction and maintained on a heated pad at 2 to 2.5% using a nose cone for the procedure. A 21G sterile needle was used to pierce through the patella tendon into the tibia. A total of 2 × 10^5^ cells suspended in 40 μl of PBS, or 40 μl of PBS control, were then injected into this space using a sterile 23G needle. After inoculation, animals were placed in a preheated recovery chamber and carefully monitored until the animal had fully regained consciousness and was mobile.

Mice were weighed weekly and bled at pretumor inoculation into a microvette 500 z-Gel tube (Sarstedt), and on cull day by cardiac puncture, and serum was separated by spinning at 10,000*g* for 5 min at 20°C. Serum was used fresh or stored at −80°C until use. Tumor burden was measured using fluorescence imaging. Animals were anesthetized using 3% isoflurane for induction, and maintained at 2 to 2.5% using a nose cone during the imaging. Animals were placed inside an IVIS Lumina LT Series III in vivo imaging system (PerkinElmer) and imaged for green fluorescence (excitation: 465 nm). Fluorescence units were measured from a 1-cm-diameter circular region of interest at the proximal tibia measured in Living Image 4.5.5 software (PerkinElmer).

### Immunohistopathology

After bone specimens were fixed in 10% formalin for a minimum of 72 hours, decalcification was performed using 10% EDTA for 10 to 14 days. The specimens were then paraffin-embedded for histopathology. Bones were cut into 5-μm sections on a microtome and mounted onto adhesive microscope slides using warm water (40°C). Sections were deparaffinized using xylene and rehydrated in a series of decreasing concentrations of alcohol. They were then immersed in citric acid buffer and steamed for 20 min. Sections were then treated with proteinase K (20 μg/ml) for 15 min and then permeabilized in 0.1% Tween for 5 min. ApopTag Red In situ Apoptosis Detection Kit (S7165 Millipore) protocol was then followed as per the manufacturer’s instructions. Before anti-digoxigenin–rhodamine was added, the sections were incubated overnight with 1:500 anti-GFP (abcam ab13970). Secondary anti-digoxigenin–rhodamine and 1:200 anti-chicken AF488 (Life Technologies A11039) were then added for 1 hour at room temperature. Sections were mounted using Diamond anti-fade (Thermo Fisher Scientific). Staining was assessed using confocal microscopy; three fields of view for each section were taken at ×20 magnification and analyzed using ImageJ (*65*).

### Statistical analysis

Statistical analysis was performed using GraphPad Prism 6 (GraphPad Software, California, USA). Experiments were performed in triplicate unless otherwise stated. Statistical significance was determined using Student’s *t* test, or for three or more groups, ANOVA was used with post hoc Dunnett’s test unless otherwise specified. Results were considered significant if *P < 0.05; **P < 0.01; ***P < 0.001; ****P < 0.0001. Data are presented as means ± SEM.

## Notes

View/request a protocol for this paper from *Bio-protocol*.
