# Supplementary material for: Metabolic profiling of prostate cancer in skeletal microenvironments identifies G6PD as a key mediator of growth and survival
Source: Sci Adv. 2022 Feb 25;8(8):eabf9096. doi: 10.1126/sciadv.abf9096 (PMC8880772; doi:10.1126/sciadv.abf9096)
Supplement: Supplementary file 1 — Figs. S1 to S8 Table S1 [file sciadv.abf9096_sm.pdf]

Science Advances
AAAS

Supplementary Materials for

Metabolic profiling of prostate cancer in skeletal microenvironments identifies G6PD as a key mediator of growth and survival

Jessica Whitburn, Srinivasa R. Rao, Emma V. Morris, Sho Tabata, Akiyoshi Hirayama, Tomoyoshi Soga, James R. Edwards, Zeynep Kaya, Charlotte Palmer, Freddie C. Hamdy, Claire M. Edwards*

*Corresponding author. Email: claire.edwards@ndorms.ox.ac.uk

Published 25 February 2022, *Sci. Adv.* **8**, eabf9096 (2022)
DOI: 10.1126/sciadv.abf9096

This PDF file includes:

Figs. S1 to S8
Table S1

Supplemental Data

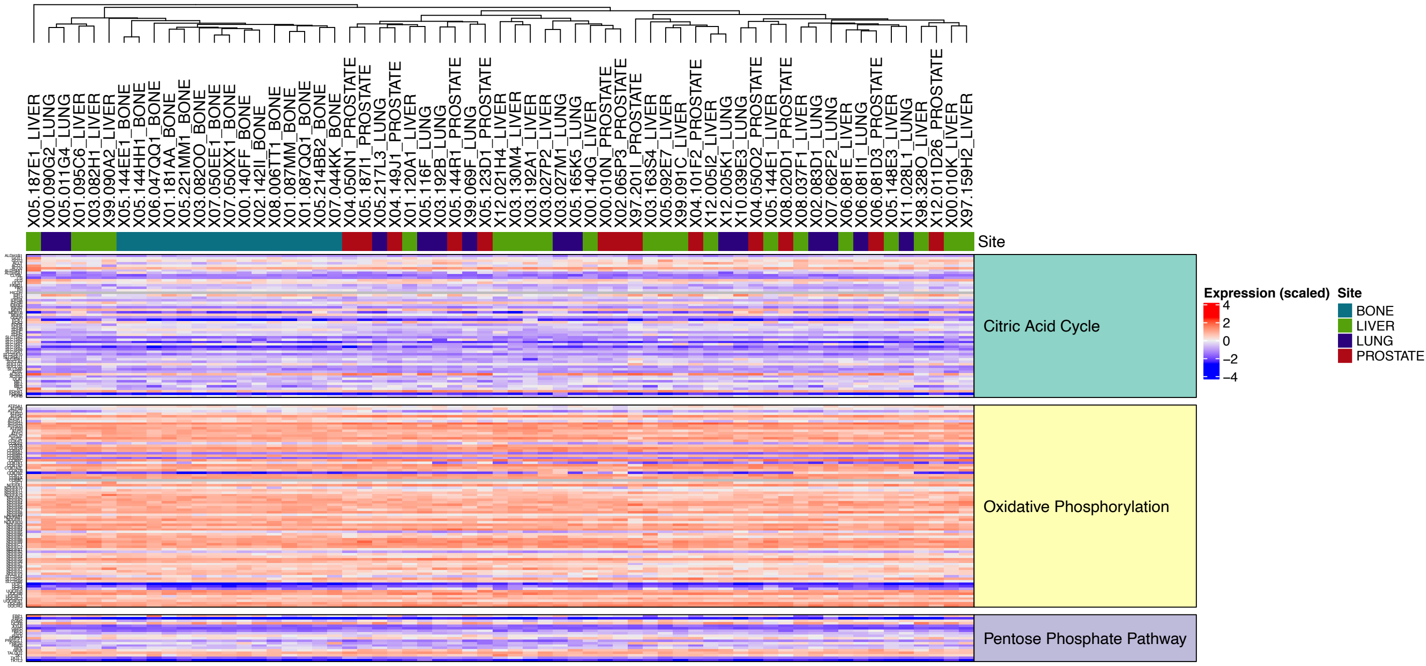

Supplemental Data Figure 1. Heatmap of mRNA expression for the indicated pathways in the Hutchinson dataset. Genes grouped by the three pathways were subset from the Hutchinson dataset using the Gaude gene set. mRNA expression data were scaled and samples (in columns) were hierarchically clustered.

Supplementary Table 1: Metabolomic analysis of PC3 prostate cancer cells cultured with HS5, ST2 or 2T3 bone stromal cells.

	ST2			HS5			2T3		
**Metabolite**	**Fold change**	**Adj p-val**	**Sig**	**Fold change**	**Adj p-val**	**Sig**	**Fold change**	**Adj p-val**	**Sig**
GSH	0.9	0.036	*	0.7	0.4855	ns	0.7	0.0992	ns
GSSG	1.2	0.0002	***	1.3	0.8735	ns	1.3	0.2841	ns
Choline	1.6	0.0009	***	0.5	0.0003	***	0.5	<0.0001	****
Betanine	1.1	0.0031	**	0.8	0.0139	*	0.8	0.0043	**
PCho	1.3	<0.0001	****	0.7	0.0006	***	0.7	0.0005	***
GPC	1.8	0.0005	***	1.3	0.0037	**	1.4	0.0017	**
Glutamate	1.1	0.0	*	0.7	<0.0001	****	0.7	0.0	***
Glutamine	1.0	0.9	ns	0.4	<0.0001	****	0.5	0.0	***
G6P	2.4	0.0	***	0.3	<0.0001	****	0.3	<0.0001	****
F6P	2.0	0.0	*	0.6	0.0	**	0.4	0.0	***
F16P	3.3	0.0	***	9.9	0.0	***	5.8	0.0	**
3PG	1.9	0.0	***	1.4	0.0	***	1.6	0.0	**
2PG	1.9	0.0	**	1.1	0.8	ns	1.3	0.2	ns
PEP	2.3	<0.0001	****	1.4	0.0	***	1.3	0.0	*
Lactate	2.1	0.0	***	2.7	0.0	***	3.2	0.0	***
DHP	3.0	0.0	***	5.5	0.0	***	2.2	0.0	**
6PG	2.2	0.0	***	0.6	0.0	***	0.9	0.1	ns
Ru5P	2.1	<0.0001	****	2.3	0.0	***	1.5	0.0	**
R5P	2.2	0.0	**	2.0	0.0	**	1.2	0.3	ns
PRPP	1.1	1.0	ns	1.5	0.0	*	0.9	0.5	ns
S7P	1.5	0.0	**	1.0	0.9	ns	0.8	0.0	**
NADP+	1.2	0.1	ns	0.7	0.0	**	0.6	0.0	***
NADPH	1.7	0.0	*	8.0	<0.0001	****	4.4	0.0	**
Acetyl-CoA	1.3	0.0	**	1.2	0.4	ns	-	-	-
Citrate	1.3	0.0	***	0.7	0.0	***	0.6	<0.0001	****
cis-Aconitate	1.3	0.0	***	0.7	0.0	***	0.6	0.0	***
Isocitrate	1.5	0.0	***	0.7	0.0	***	0.7	0.0	***
2-oxoglutarate	1.6	0.0	**	1.7	0.0	**	1.2	0.0	**
Succinate	1.5	0.0	***	0.7	0.1	ns	0.8	0.0	*
Fumarate	1.5	0.0	***	1.2	0.0	**	1.3	0.0	***
Malate	1.4	<0.0001	****	1.1	0.0	**	1.1	0.0	*

PC3 cells were co-cultured for 24 hr with HS5, ST2 or 2T3 bone stromal cells and metabolite levels measured with CE-MS. Fold change as compared with prostate cancer cells cultured alone. Two-way Anova and Dunnett's Multiple Comparison Test.

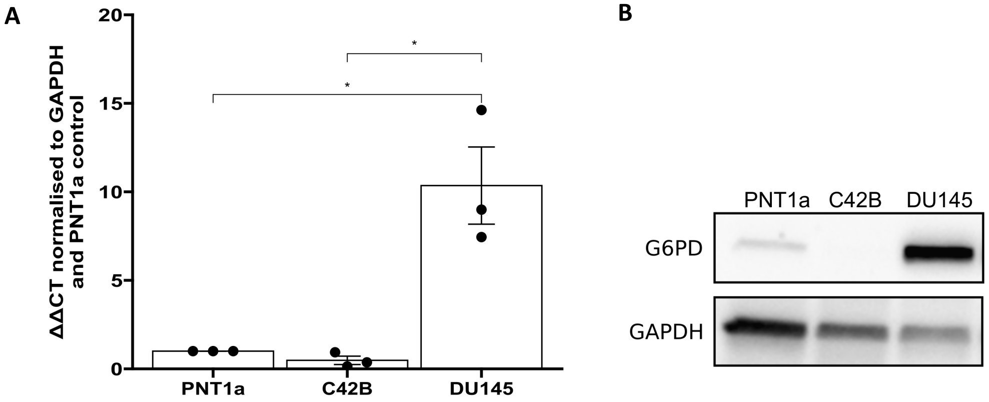

Supplemental Data Figure 2. Expression of G6PD in C4-2B and DU145 prostate cancer cells. (A) mRNA expression of G6PD in PNT1a, C42B and DU145 prostate cancer cells. Statistical test used = one-way ANOVA with post-hoc Tukey's test. *=p<0.05, **=p<0.01. Error bars=SEM. (B) Protein expression of G6PD in PNT1a, C42B and DU145 prostate cancer cells.

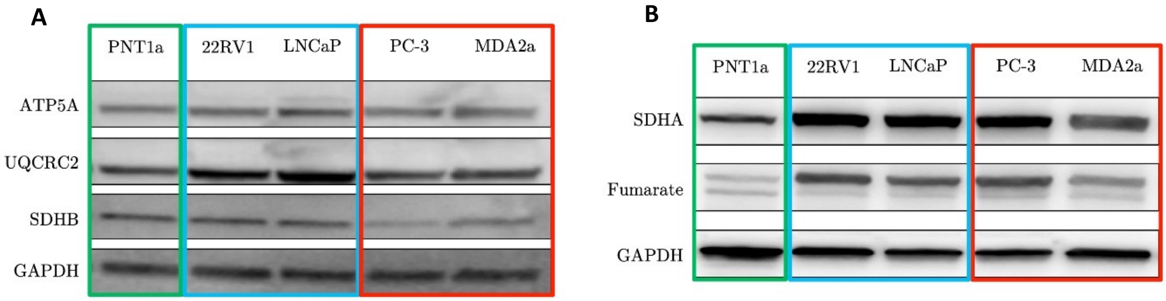

Supplemental Data Figure 3. Expression of OXPHOS complexes and TCA enzymes in prostate cancer cells. (A) Protein expression of OXPHOS complexes. ATP5A: ATP synthase F1 subunit alpha; UQCRC2: ubiquinol-cytochrome C reductase core protein 2; SDHB: succinate dehydrogense (ubiquinone) iron-sulphur subunit. **(B)** Protein expression of TCA enzymes. SDHA: succinate dehydrogenase complex flavoprotein subunit A.

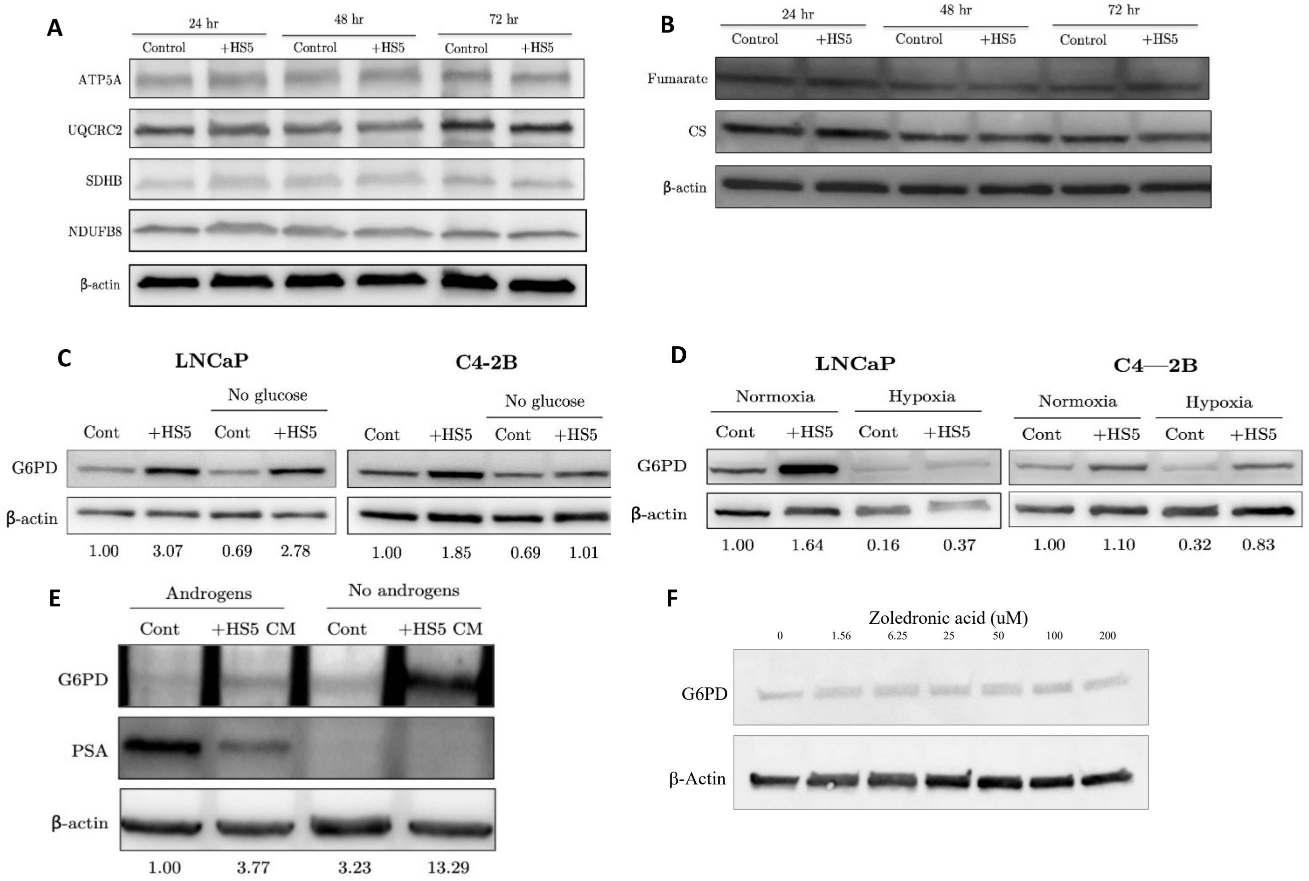

Supplemental Data Figure 4. Altered expression of metabolic proteins in prostate cancer cells in response to bone marrow stromal cells. (A) Protein expression of OXPHOS complexes in LNCaP after transwell co-culture with HS5 cells for the specified time points. (B) Protein expression of TCA enzymes (CS= citrate synthase; βA = beta-actin) in LNCaP after transwell co-culture with HS5 cells for the specified time point. (C) G6PD protein expression in LNCaP and C4-2B cells after 72 hr single or co-culture with HS5 BMSC in normal or glucose free RPMI. Densitometry quantification of G6PD band normalised to β-actin and control shown below blot. (D) G6PD protein expression in LNCaP and C4-2B cells after 72 hr single or co-culture with HS5 BMSCs in normoxic or hypoxic conditions. Densitometry quantification of G6PD band normalised to β-actin and control shown below blot. (E) Western blot showing G6PD and PSA protein expression in LNCaP cells after 72 hr treatment with androgen-free media +/- 50% HS5 androgen-free CM. Densitometry quantification of G6PD band normalised to β-actin and control shown below blot. (F) PC3 prostate cancer cells treated with increasing concentrations of zoledronic acid for 24h. Blots representative of 3 biological repeats.[1]

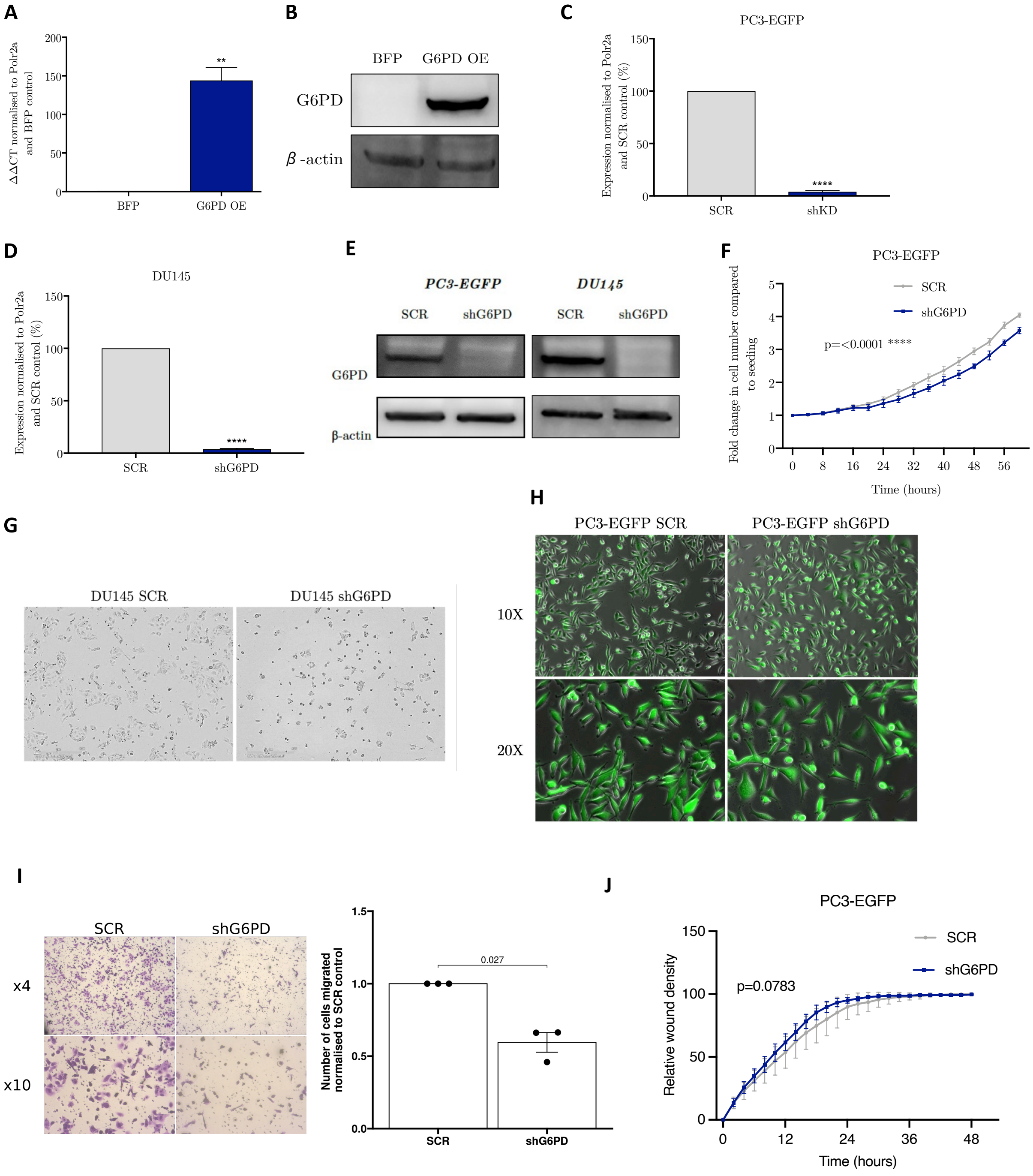

Supplemental Data Figure 5. Effect of G6PD overexpression and knockdown. (A) Taqman qPCR confirming G6PD overexpression. (B) Western blot confirming G6PD overexpression. Blot representative of 3 biological repeats. Statistical test = Student t-test. Error bars = SEM. ** p=<0.01. Error bars = SEM. Stable knockdown was performed in PC3-EGFP and DU145 cells and confirmed at the mRNA (C, D), and at the protein level (E). Western blot representative of 3 biological repeats. Statistical test used = Student's t-test. **** p=<0.0001. Error bars = SEM. (F) PC3 proliferation measured using an IncuCyte live cell imaging system. Statistical test used = two way ANOVA, column factor p-value shown. (G) DU145 SCR and shG6PD cells; 10X images taken on an IncuCyte live cell analysis imaging system. (H) PC3-EGFP SCR and shG6PD cells. Merged images of brightfield and GFP. Images taken on a Nikon Eclipse TE300 inverted microscope. (I) Transwell migration assay showing DU145 migration through a transwell membrane at 24 hr and the number of cells migrated normalised to SCR control. Statistical test = Student's t-test. (J) Scratch assay showing relative scratch wound confluency in PC3 cells Statistical test = two-way ANOVA.

A

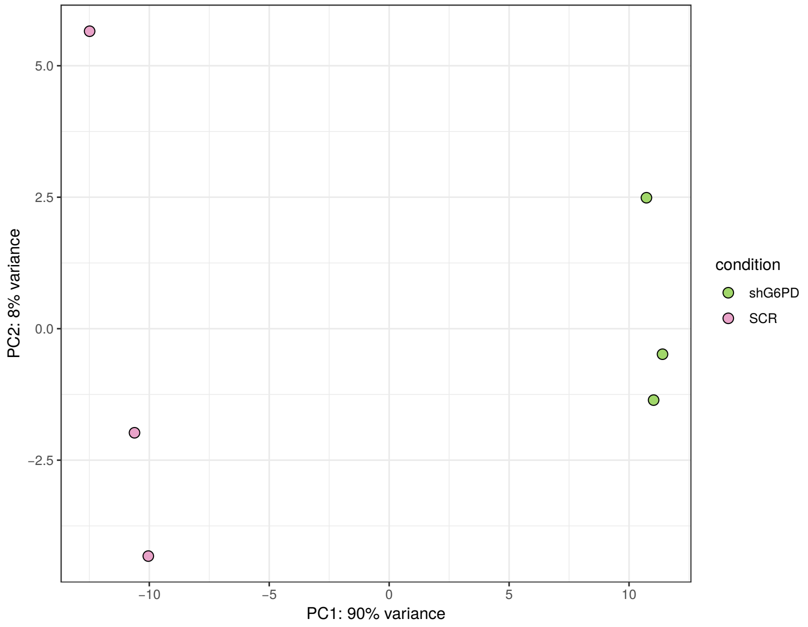

B

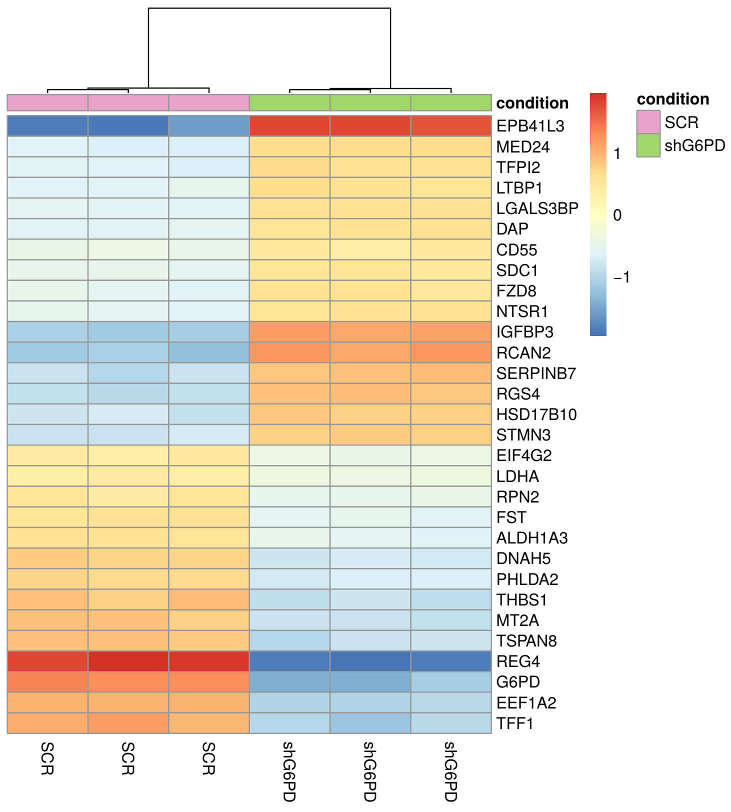

Supplemental Data Figure 6. Transcriptomic profiling of G6PD knockdown PC3 prostate cancer cells. (A) PCA plot from RNA Seq of PC3-EGFP SCR and shG6PD cells. (B) Heatmap of 30 most differentially expressed genes. N=3.

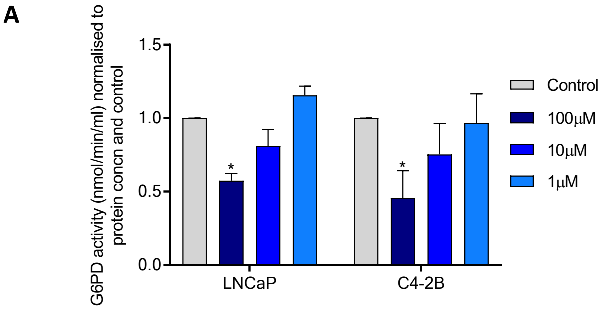

Supplemental Data Figure 7. Pharmacological inhibition of G6PD with 6AN. G6PD activity in C4-2B (N=4) and LNCaP (N=2) cells after 24 hr treatment with varying doses of 6AN. Statistical test = one-way ANOVA with Dunnett's post hoc test.

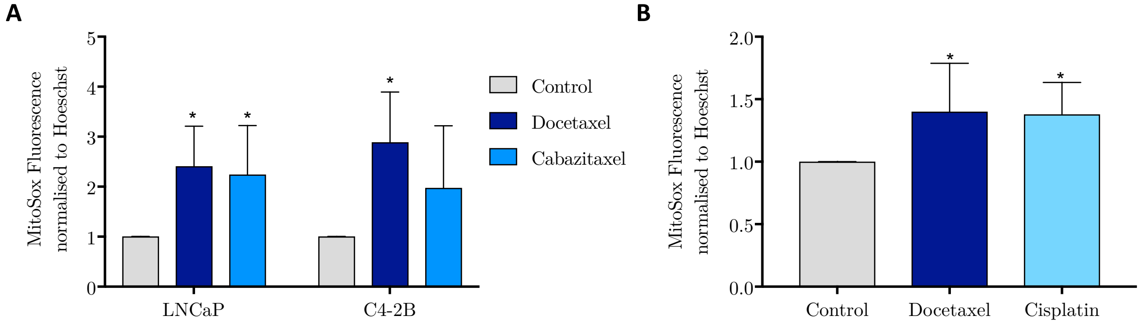

Supplemental Data Figure 8. Chemotherapy increases mitochondrial ROS. (A) MitoSox fluorescence measured after 72 hr treatment with 10nM docetaxel or 10μM cabazitaxel in LNCaP or C4-2B cells. (B) MitoSox fluorescence measured after 72 hr treatment with 10nM docetaxel or 10μM cisplatin in PC3 cells. Statistical test used = Student's t-test compared to untreated control. * p=<0.05. Error bars = SEM.
